# Estimating Active Transportation Behaviors to Support Health Impact Assessment in the United States

**DOI:** 10.3389/fpubh.2016.00063

**Published:** 2016-05-02

**Authors:** Theodore J. Mansfield, Jacqueline MacDonald Gibson

**Affiliations:** ^1^Department of Environmental Sciences and Engineering, Gillings School of Global Public Health, University of North Carolina at Chapel Hill, Chapel Hill, NC, USA

**Keywords:** transportation, walking, environment and public health, health impact assessment, environment design

## Abstract

Health impact assessment (HIA) has been promoted as a means to encourage transportation and city planners to incorporate health considerations into their decision-making. Ideally, HIAs would include quantitative estimates of the population health effects of alternative planning scenarios, such as scenarios with and without infrastructure to support walking and cycling. However, the lack of baseline estimates of time spent walking or biking for transportation (together known as “active transportation”), which are critically related to health, often prevents planners from developing such quantitative estimates. To address this gap, we use data from the 2009 US National Household Travel Survey to develop a statistical model that estimates baseline time spent walking and biking as a function of the type of transportation used to commute to work along with demographic and built environment variables. We validate the model using survey data from the Raleigh–Durham–Chapel Hill, NC, USA, metropolitan area. We illustrate how the validated model could be used to support transportation-related HIAs by estimating the potential health benefits of built environment modifications that support walking and cycling. Our statistical model estimates that on average, individuals who commute on foot spend an additional 19.8 (95% CI 16.9–23.2) minutes per day walking compared to automobile commuters. Public transit riders walk an additional 5.0 (95% CI 3.5–6.4) minutes per day compared to automobile commuters. Bicycle commuters cycle for an additional 28.0 (95% CI 17.5–38.1) minutes per day compared to automobile commuters. The statistical model was able to predict observed transportation physical activity in the Raleigh–Durham–Chapel Hill region to within 0.5 MET-hours per day (equivalent to about 9 min of daily walking time) for 83% of observations. Across the Raleigh–Durham–Chapel Hill region, an estimated 38 (95% CI 15–59) premature deaths potentially could be avoided if the entire population walked 37.4 min per week for transportation (the amount of transportation walking observed in previous US studies of walkable neighborhoods). The approach developed here is useful both for estimating baseline behaviors in transportation HIAs and for comparing the magnitude of risks associated with physical inactivity to other competing health risks in urban areas.

## Introduction

Physical inactivity is a leading cause of premature mortality in the United States, contributing to an estimated 234,000 premature deaths annually (1). In addition, physical inactivity is associated with increased risk for chronic diseases including type 2 diabetes, cardiovascular disease, and colon cancer (2–4). Recognizing the risks associated with physical inactivity, the Centers for Disease Control and Prevention (CDC) recommends that individuals accrue a minimum of 150 min of moderate intensity physical activity per week (5). One important source of physical activity is walking and biking for transportation (known as “active transportation”). For example, a study of respondents to the National Household Travel Survey (NHTS) found that the median time spent walking to or from public transit among individuals who use public transportation was 21 min per day (6).

Transportation agencies in the United States are increasingly recognizing the importance of active transportation in pursuit of broader public health goals (7, 8). To support the incorporation of health considerations into decision-making in sectors such as transportation, health impact assessment (HIA) has emerged in recent years. A number of recent transportation HIAs have sought to estimate the health impacts of investments that support walking and biking for transportation (9). However, active transportation HIAs are often conducted with limited data. While a large body of work has linked active transportation behaviors to characteristics of the built environment, such as population density, the diversity of land uses, and access to public transit (10), baseline data on walking and biking for transportation are not routinely available at the local level. Baseline active transportation data are important in targeting interventions to increase transportation physical activity and are essential in estimating the expected population-level health benefits of infrastructure and other investments to promote active transportation. Lacking readily available baseline data on walking and biking behaviors, active transportation HIAs must rely on potentially inaccurate estimates or costly primary data collection, the latter of which often is not possible within the budget of the HIA.

While baseline active transportation data are scarce at the local level, a number of US national surveys collect data on transportation behaviors. However, a recent CDC summary of these surveys revealed differences in methods used, geographic scale, and estimates of active transportation (11).

Travel and time-use surveys, including the NHTS and the American Time Use Survey, contain detailed travel information, including the frequency of walking and biking trips for different purposes, but only for a single day (12, 13). Both the National Health and Nutrition Examination Survey and the National Health Interview Survey assess habitual physical activity behaviors, including walking and biking for transportation, and ask respondents to recall activity over the previous week (14, 15). The American Community Survey (ACS) collects data on typical mode of transportation to work, including walking and biking, but does not gather information from respondents regarding typical walking and biking duration (16).

The geographic scale of surveillance also varies greatly across surveys. While large national surveys such as the NHTS offer great detail at the individual level, geographic resolution is limited. Conversely, the ACS offers much greater spatial resolution but limited information at the individual level.

Due to the differences in methods and scales across currently available surveys, estimates of the prevalence of walking and biking for transportation in the US population vary widely: in the the 2012 ACS, which captures only active commuting behaviors, 3.4% or respondents reported walking or biking to work. Conversely, 31.4% of respondents reported some walking or biking in the previous week in the 2011–2012 National Health and Nutrition Examination Survey, which captures all active transportation behaviors (11). Nonetheless, the NHTS and ACS collect a number of shared variables, including individual demographic characteristics, typical transportation mode to work, and basic built environment metrics (12, 16). These shared variables provide an opportunity to use the NHTS and ACS in tandem to offer a more detailed understanding of walking and biking for transportation at fine spatial resolution.

To address the gap in understanding the influence of transportation choices on physical activity, we use data from the 2009 NHTS to develop a statistical model that estimates weekly time spent walking and biking for adults in the US as a function of demographic and built environment variables routinely collected in the ACS. We then validate the model using data from a separate household travel survey conducted in the Raleigh, NC, USA, metropolitan area. We demonstrate how the statistical models can be combined with readily available ACS data to estimate baseline active transportation time across the Raleigh–Durham–Chapel Hill, NC, USA, region. Finally, we illustrate how the statistical model could be used to support transportation-related HIAs by applying the model to estimate the health impacts of multiple hypothetical scenarios in which changes to the built environment increase transportation physical activity.

## Materials and Methods

Data from the 2009, NHTS were used to estimate a set of regression models: daily walk and bike trip count models, trip purpose probability models, and trip duration models. These models were estimated separately for walk and bike trips for working and non-working adults. These models were then combined to estimate weekly walking and biking time based on individual and built environment data from the ACS. Statistical analysis was performed using Stata 13 (College Station, TX, USA), and the model was applied in the study region using Analytica 4.3 (Los Gatos, CA, USA).

### National Household Travel Survey

The NHTS, last administered in 2009, collects travel information from households across United States. Household, personal, and vehicle characteristics are collected via an initial telephone interview. Subsequently, participants use a travel diary to record all travel for an assigned day, and these travel data are collected in a follow-up phone interview. The 2009 dataset contains information on 1,116,321 trips taken by 308,901 individuals living in 150,147 households and is organized into four files (household file, person file, day trip file, and vehicle file). The data are weighted to match national demographic characteristics.

#### Data Preparation

To prepare the 2009 NHTS data for our purposes, we first summed walk and bike trip counts in the day trip file for each individual in the person file and generated two new variables to store walk and bike trip counts in the person file. We then collapsed commute mode to work and trip mode data into four categories: private vehicle (including all vehicle types and carpool), public transit (including fixed-route and paratransit), walk, and bike. In the day trip file, trip purpose was collapsed into five categories (work, shopping, social, recreational, and personal/family business), using roundtrip purpose definitions (the 1990 trip purpose definitions variable). Race and Hispanic status were combined into a single race/ethnicity variable (Hispanic, non-Hispanic White, non-Hispanic Black, non-Hispanic Asian, and non-Hispanic other). The month variable was collapsed into four seasons, and a weekend dummy variable was generated using the travel day of week variables. Finally population density was divided by 1,000. We then merged the person and day trip data files as described in the NHTS supporting documentation (17). The data were then stratified into two sub-groups: working adults (individuals aged 18 and over who report working in the previous week) and non-working adults (individuals aged 18 and over reporting no work in the previous week).

#### Outliers

**Figure 1:**
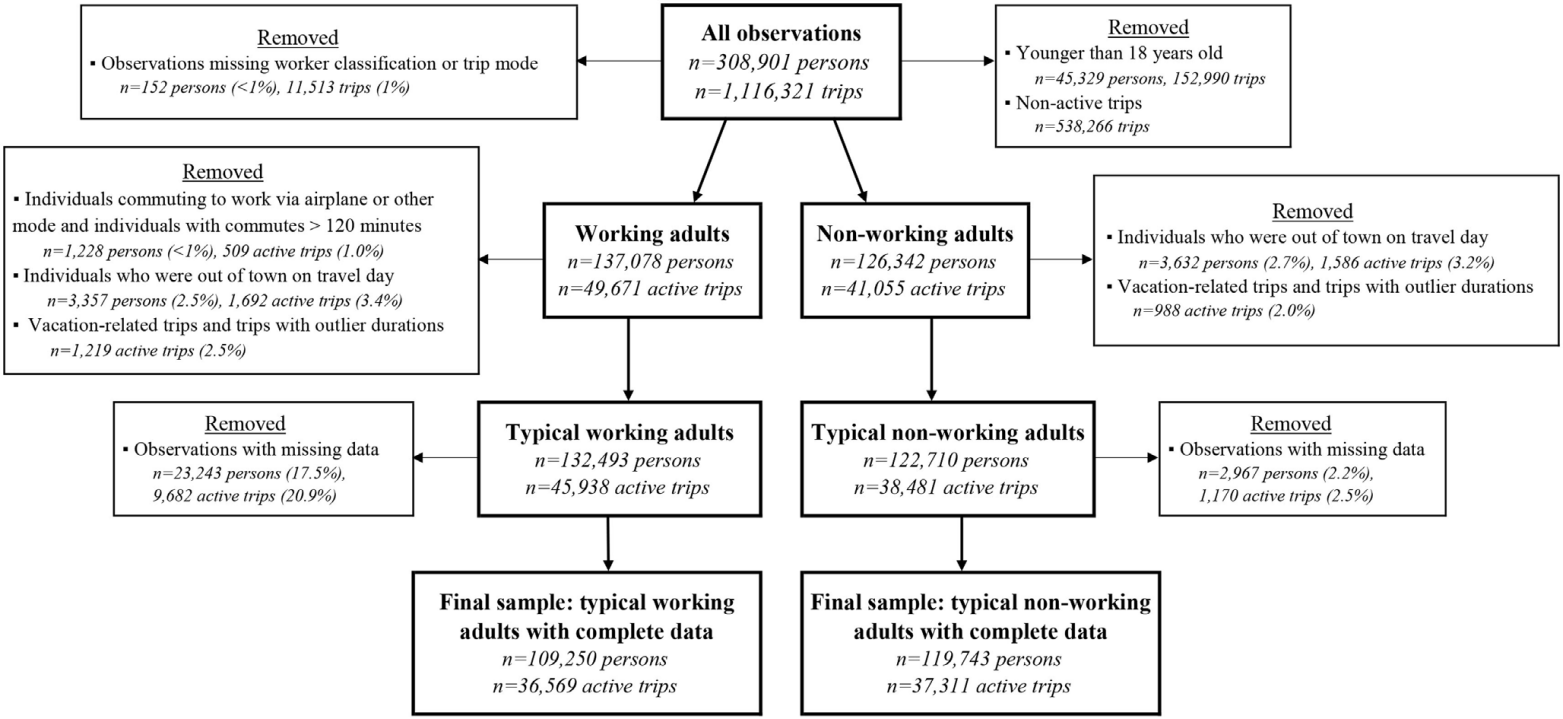
**Flowchart illustrating data cleaning and stratification of the 2009 NHTS dataset into working and non-working adults**.

Because we focus on routine active travel among adults, we removed observations from the NHTS that do not represent typical transportation behaviors. In the person file, we dropped individuals who reported being out of town when the survey was administered, commuting to work via airplane or “other” travel modes, or having work commutes lasting longer than 2 h. From the trip file, we dropped all non-active trips, vacation-related trips, and trips with durations in the highest 1% of the mode-specific trip duration distributions. In total, we removed 4,585 persons and 3,420 active trips from the sample of working adults and 3,632 persons and 2,574 active trips from the sample of non-working adults due to atypical responses (Figure 1).

#### Missing Data

We dropped observations from the person file if race, education, presence of a medical condition restricting travel variables, or commute mode to work (for working adults only) were missing. Due to missing data, we removed 23,243 persons and 9,682 active trips from the sample of working adults and 2,967 persons and 1,170 active trips from the sample of non-working adults. Commute mode to work was the most common missing variable (15.9% of the remaining sample) due to a skip in the survey questionnaire triggered when the respondent reported not traveling to work in the previous week, potentially indicating that the week was atypical for that individual.

After removing atypical transportation behaviors and observations with missing, the final sample of working adults contained 45,938 trips made by 109,250 persons, and the final sample of non-working adults contained 37,311 trips made by 119,743 persons (Figure 1). Descriptive statistics of the final sample are presented in the Tables S1 (Person File) and S2 (Trip File) in Supplementary Material.

### Transportation Physical Activity Estimation Framework

To estimate weekly time spent walking and biking for transportation, count models were first used to estimate the number of walk and bike trips taken by an individual during a typical day (see Daily Trip Count models). Because trip duration in the NHTS varies significantly with trip purpose, the distribution of trips among different purposes is also an important factor in estimating total transportation physical activity. Multinomial logistic regression models were used to predict the probability that a given walk or bike trip was for one of five purposes: (1) commuting to work; (2) shopping; (3) socializing; (4) engaging in recreation; or (5) tending to personal or family business (see Trip Purpose Probability Models). Finally, trip duration was estimated for each trip purpose (see Trip Duration Models). Estimated trip counts were combined with trip purpose probabilities and purpose-specific duration estimates to predict daily walking and biking time for individuals using Eq. 1:

(1) $${\text{TT}}_{m,i}=\overset{5}{\underset{p=1}{\sum }}\left(E\left(t_{m,i}\right)\times \left(Pr(p_{m,i})\times d_{p,m,i}\right)\right)$$

in which TT*_*m,i*_* is daily minutes spent traveling using mode *m* for individual *i*, *E*(*t_*m,i*_*) is the expected daily number of trips take using mode *m* for individual *i*, Pr(*p_*m*_*) is the probability that a trip taken by individual *i* using mode *m* is for purpose *p*, and *d_*p,m*_* is trip duration for a trip taken by individual *i* for purpose *p* using mode *m*.

Walking and biking time were combined by multiplying each activity by its intensity, measured by metabolic equivalents (METs). METs measure the intensity of physical activity relative to an individuals’ resting metabolic rate, which is equal to one MET. By multiplying the intensity of an activity by its MET value and its duration, total physical activity dose from a variety of activities with differing intensities may be calculated, expressed in METs multiplied by the duration of the activity to obtain MET-hours. Walking and biking for transportation have MET values of 3.5 and 6.8, respectively (18). Equation 1 was thus used to transform biking and walking time into a daily physical activity dose:

(2) $${\text{TPA}}_{i}=\frac{\left({\text{TT}}_{m=\text{walk,}i}\times 3.5\right)+\left({\text{TT}}_{m=\text{bike,}i}\times 6.8\right)}{60\text{min/h}}$$

in which TPA_*i*_ is daily physical activity from walking and biking for individual *i* in MET-hours, TT_*m*=walk, *i*_ is daily time spent walking for transportation for individual *i* in minutes, and TT_*m*=bike,*I*_ is daily time spent biking for transportation for individual *i* in minutes.

The following sections describe the three regression models used to estimate *E*(*t_*m*_,_*i*_*), Pr(*p_*m,i*_*), and *d_*p,m,i*_*. For all models, explanatory variables included both individual characteristics (commute mode to work, age, sex, and race) and built environment variables reported in the NHTS (population density and proportion of housing units that are rented in the block group in which the individual resides). Commute mode to work is intuitively related to active transportation behavior. Age, sex, and race are associated with transportation walking and biking (19). Population density has a well-documented relationship with walking and biking for transportation (13). Finally, percent of rental units may be a rough proxy for land-use diversity, also strongly linked to walking and biking for transportation (13). All models included controls for educational attainment, travel day of the week (weekday or weekend), the season in which the survey was administered, whether or not the respondent reported having a medical condition that may restrict travel, whether the interview was conducted with a proxy respondent, whether the metropolitan statistical area in which the respondent resided had heavy rail (which may influence urban form and trip-making in unique ways), and state, Census division, or Census region fixed effects. In all regression models, variables were retained if significant at the 10% level.

#### Daily Trip Count Models

Daily walk and bike trip count data contained high proportions of zeroes and displayed little evidence of overdispersion (Figure S1 in Supplementary Material). Specification tests (Vuong and Lagrange multiplier) were used to select an appropriate form for the daily trip count models (20). These specification tests revealed very strong (*p* < 0.001) evidence for zero-inflated Poisson models to represent both walk and bike trip counts for working and non-working adults (Figure S2 and Tables S3 and S4 in Supplementary Material). Thus, daily walk and bike trip counts were estimated using the following model (21):

(3) $$\begin{array}{cc} \text{Pr}\left(Y_{i}=y_{i}|x_{i}\right) & =\{\begin{array}{cc} \pi_{i}+\left(1-\pi_{i}\right)e^{-\lambda_{i}}, & \mathrm{if}\;y_{i}=0\\ \frac{\left(1-\pi_{i}\right)e^{-\lambda_{i}}\lambda_{i}{}^{y_{i}}}{y_{i}!}, & \mathrm{if}\;y_{i}>0 \end{array}\\ & \;\lambda_{i}=e^{\left(\alpha +x_{i}^{T}\beta \right)} \end{array}$$

where π_*i*_ is the probability that daily walk or bike trip counts always equals zero, ***x***_*i*_ is a vector of individual-specific regressors, and ***β*** is a vector of regression coefficients. Variables were retained in the model if significant at the 10% level and robust SEs were used.

#### Trip Purpose Probability Models

Multinomial logistic regression models were used to predict the probability of different trip purposes based on individual characteristics and built environment variables. Accordingly, the probability that a trip is for purpose *j* is expressed as (20):

(4) $$\text{Pr (}y_{i}=p\text{)}=\frac{e^{\left(x_{i}^{T}\beta \right)}}{1+\sum_{p=1}^{P-1}e^{\left(x_{i}^{T}\beta \right)}},\;for\;p=1,\ldots ,\;P-1$$

where Pr(*y_*i*_* = *p*) is the probability of trip purpose *p* for individual *i*, *P* is the number of outcomes (in this case, five: work commute, shopping, social, personal/family business), ***x***_*i*_ is a vector of individual-specific regressors, and *β* is a vector of regression coefficients.

#### Trip Duration Models

Generalized estimating equation (GEE) models with a log link were used to estimate trip duration based on individual characteristics and built environment variables. Because an individual may take multiple trips during the day and trip characteristics may be correlated within and across individuals, the data are treated as a panel of individuals observed taking multiple trips. GEE models offer a robust approach to estimating SEs when using data that are correlated within clusters of observations (in this case, the relatedness of trips within individuals) (22). Trip duration may be influenced by different factors depending on trip purpose; thus, commute mode to work, travel time to work, population density, and percent rental units were interacted with trip purpose in trip duration models for working adults. Population density and percent rental units were interacted with trip purpose in trip duration models for non-working adults. These models may be expressed as (20):

(5) $$g\;(d_{m,i})=x_{i}^{T}\beta \;\;$$

where *d_*m,i*_* is trip duration for individual *i* using mode *m*, *g*(*d_*m,i*_*) is the link function, $x_{i}^{T}$ is a vector of trip-specific regressors, and ***β*** is a vector of estimated coefficients.

#### Marginal Effects

Average marginal effects of explanatory variables for each regression model (count, trip purpose, and trip duration) were estimated using the margins command in Stata. To calculate the combined marginal effect of explanatory variables on daily walking and biking time, a model was developed in Analytica that incorporated estimated regression coefficients for each model into Eq. 1. Monte Carlo simulation was used to develop SEs for combined marginal effects.

#### Model Validation

To validate model performance, model predictions were compared to results from a 2006 household travel survey conducted in the Raleigh–Durham–Chapel Hill metropolitan area as part of routine transportation planning (23). Survey respondents provided demographic information and recorded all trips for 1 week day. The full validation dataset contained 6,618 workers. We dropped 3,427 individuals due to missing data, largely due to missing race/ethnicity (*n* = 2,789). We then calculated observed daily MET-hours for all individuals with complete data in the validation dataset from their recorded trips using Eq. 2. Finally, we used Eq. 1 to estimate daily MET-hours for the validation survey (TPA_*i*,est_) sample and compared model predictions to observed values (TPA_*i*,obs_).

Descriptive statistics for the validation sample are presented in the Tables S1 and S2 in Supplemental Material. Compared to the NHTS, respondents in the validation survey reported fewer total walk and bike trips. The validation sample also has higher education levels, fewer proxy respondents, and only contains responses from the winter and spring. However, most differences between the two datasets are included as controls in the NHTS regression models.

### Applying the Model to Estimate Physical Activity for Population Subgroups

To estimate weekly transportation physical activity across the Raleigh–Durham–Chapel Hill metropolitan region, we first used Eq. 1 to estimate *TPA_*i*_* for all possible combinations of variables that vary on the individual level and across block groups in the study area. We excluded recreational trip durations when summing total walking and biking time in Eq. 1 to focus on purpose-oriented (non-recreational) transportation physical activity. Four of these variables – commute mode to work *c* (including a category for non-workers), age *a*, sex *s*, and race/ethnicity *r* – vary on the individual level. The fifth variable, *g*, represents the combined effect of all variables and controls that are measured at the block group – population density, percentage of units that are rentals, travel time to work by mode, and educational attainment. Population density was calculated using block-group population counts obtained from the 2013 ACS and area obtained from Census TIGER files (24, 25). If household income and/or travel time to work data were missing at the block group level due to sampling limitations, tract-level data were used instead. If tract-level data were also missing, county-level data were used. In the block-group level Census data, time to work for bicyclists is combined with other modes (motorcycle, taxicab, and other). If the reported travel time to work by bicycle, motorcycle, taxicab, and other modes was greater than the travel time reported for private vehicles, the lower of these values was used. Missing data were treated as described above, still using the lower value if travel time reported at the tract or county level exceeded motor vehicle travel time.

Equation 1 was used to estimate *TPA_*i*_* for a typical weekday and for a typical weekend day for all possible unique combination of *c*, *a*, *s*, *r*, and *g*. Weekly estimates were then obtained by multiplying the typical weekday estimate by five and typical weekend estimate by two, and then summing the products. These estimates were stored in a five-dimensional matrix, **TPA**. This matrix contained approximately four million cells, each containing a unique estimate of *TPA_*i*_* associated with 1 of 5 possible commuting behaviors, 1 of 96 possible ages, 1 of 2 sexes, 1 of 5 race/ethnicities, and 1 of 835 block groups. To reflect the uncertainty of regression coefficients, **TPA** was estimated using Monte Carlo simulation in Analytica. The SD of each estimate was stored in a second matrix, **TPA_SD_**, with the same dimensions as the matrix **TPA**. **TPA**_SD_, was used to model uncertainty and generate 95% confidence intervals for our estimates using Monte Carlo simulation in Analytica.

### Applying Physical Activity Estimates to the Population

Once the matrix **TPA** was generated, data from the 2013 ACS were used to develop joint distributions of population characteristics across the four individual dimensions (*c*, *a*, *s*, and *r*) for each block group in the study area. To do so, the normalized distribution of age by sex was first multiplied by age- and gender-specific labor force participation functions to define the age and sex distribution of workers and non-workers in each block group. Labor force participation rates by sex for each county were taken from the 2013 ACS (24). These data were smoothed over age by fitting fourth-order splines to the raw data for men and women in each county (Table S3 and Figure S6 in Supplementary Material). Then, the distribution of workers was multiplied by the distribution of reported commute mode to work, creating the five dimensions of *c* noted previously (private vehicle, transit, walk, bike, and not in labor force). Finally, this distribution was multiplied by the distribution of the population by race/ethnicity in each block group. When performed for all block groups in the study region, this process yielded a matrix **NPD** that contained normalized distributions of the populations in each block group across the same dimensions as **TPA**. Finally, **NPD** was multiplied by a vector **P** containing the aggregate population of each block group in the region. This process resulted in a representation of block group populations distributed across age, sex, race/ethnicity, and commute mode to work (including a category for non-workers) based on the 2013 ACS (24). An example of this procedure for a single block group is provided in the Supplementary Material.

### Health Impact Estimates

We estimated health benefits of walking and biking in the study region by comparing predicted transportation physical activity to a counterfactual scenario in which individuals walked 37.4 min per week for transportation – the average level of walking observed in groups of high- and low-income walkable neighborhoods in Baltimore and Seattle (26). This calculation requires an estimate of the relative risk of all-cause mortality as a function of transportation physical activity, denoted as RR_M_(TPA). According to a recent meta-analysis (27), this dose–response function can be estimated as:

(6) $${\text{RR}}_{\text{M}}(\text{TPA})=0.90^{\left(\frac{\text{TPA}}{11.25\text{MET}-\text{hours}}\right)}$$

The fractional change in mortality under the counterfactual scenarios, in comparison to current conditions, was estimated from:

(7) $${\text{AF}}_{\text{TPA}}=\frac{\left(\begin{array}{l} \int_{\text{TPA}=0}^{\infty }\left(1-RR_{M}\left(\text{TPA}\right)\right)f_{\text{est}}\left(\text{TPA}\right)d\text{TPA}-\\ \int_{\text{TPA}=0}^{\infty }\left(1-RR_{M}\left(\text{TPA}\right)\right)f_{\text{cf}}\left(\text{TPA}\right)d\text{TPA} \end{array}\right)}{1+\int_{\text{TPA}=0}^{\infty }\left(1-RR_{M}\left(\text{TPA}\right)\right)f_{\text{est}}\left(\text{TPA}\right)d\text{TPA}}$$

where AF_TPA_ is the fraction of mortality avoidable by additional active transportation in the study region, *f*_est_(TPA) is the current probability distribution of transportation physical activity as estimated in Eq. 2, and *f*_cf_(TPA) is a probability distribution of transportation physical activity in the counterfactual scenario (28, 29). Finally, the total change in mortality was calculated as follows:

(8) $${\text{AM}}_{\text{TPA}}={\text{DR}}_{b}\times {\text{AF}}_{\text{TPA}}$$

where AM_TPA_ is avoided mortality due to active transportation, and DR_*b*_ is the age- and sex-specific baseline death rate for each county in the study region, taken from the North Carolina State Center for Health Statistics (30). To alleviate the small number problem (i.e., age groups with no observed deaths in a given year), a 5-year average death rate was calculated for males and females for each age group in each county (Table S6 in Supplementary Material). Equations 7 and 8 were employed across the same dimensions as **TPA**; thus, health impact estimates may be stratified by age, sex, race/ethnicity, commute mode to work, and block group or any combination of these dimensions. The World Health Organization suggests applying Eq. 6 only for bicyclists between the ages of 20 and 64 and walkers between the ages of 20 and 74 (31). Thus, we restricted our calculation of health impacts to these age ranges.

### Hypothetical HIA Application

To illustrate how our regression models could be applied to support active transportation HIA, we estimated health benefits for three hypothetical interventions to support increased walking and biking for transportation. A recent meta-analyses-derived elasticities linking changes in the built environment to changes in transportation behavior (10). According to this meta-analysis, five built environment dimensions – land use density, land use diversity, physical design, access to transit, and access to destinations – can affect transportation behavior and, in turn, transportation physical activity. For example, a 1% increase in the number of intersections per square mile is associated with a 0.39% increase in walking. Similarly, 1% increases in land use diversity and the number of transit stops per square mile are each associated with 0.15% increases in walking. A 1% increase in transit stop coverage also is associated with increasing transit use by 0.29%. In the first scenario, we assume that land-use diversity, transit stop coverage, and intersection density all increase by 10% across the study region, resulting in a 7.9% increase in walking for the entire population. For the second scenario, we assume that the same built environment changes result in 7.9% of current drivers walking instead of driving to work. In the third, we assume that transit coverage increases by 50% across the study region, resulting in 14.5% of current drivers switching to public transit for their work commutes. We then used Eqs. 7 and 8, replacing *f*_cf_(TPA) with the new counterfactual distributions of transportation physical activity.

## Results

### Number of Walking and Biking Trips

To estimate the influence of means of transportation to work, individual characteristics, and built environment variables on the number of daily walking and biking trips, we fitted zero-inflated Poisson regression models to data from the 2009 NHTS. Results show that those who walk, bike, or take public transit to work are significantly more likely to be in the ‘‘not always zero’’ daily walk trip count group, compared to those who drive to work (Table 1, logistic model). This effect is the strongest for those walking to work (OR = 16.6) and also quite strong for those riding transit to work (OR = 4.73). Additionally, among individuals walking at least once per day, those who walk to work take 1.68 times as many walk trips as those commuting by private vehicle (Table 1, count model). Increased population density and percentage of housing units that are rented are both associated with a slightly higher probability of taking at least one walk trip and higher walk trip counts among those who walk at least once per day. For non-working adults, population density and percentage rental units are significantly associated with both increased likelihood of being in the ‘‘not always zero’’ daily walk trip count group and, for individuals in the “not always zero” group, increased daily walk trip counts. In sum, walk trip count models show that individuals who walk, ride transit, or, to a lesser extent, bike to work are likely to take more walk trips than those who drive to work. Increased population density and percentage of rental units both have additional significant, albeit small, impacts on daily walk trip counts.

**Table 1:** **Model for estimating daily number of walking trips**.

Variable		Odds ratio	
		Working adults^a^	Non-working adults^a^
Logistic model (probability not always zero)	Mode to work		
	Private vehicle	(*ref*)	–
	Public transit	4.73***	–
	Walk	16.6***	–
	Bike	2.00**	–
	Population density	1.01**	1.03***
	Percent rented	1.01***	1.01***
	Age	1.02**	0.99***
	Age squared	0.9997**	–
	Race/Ethnicity		
	Non-Hispanic White	(*ref*)	(*ref*)
	Non-Hispanic Black	0.64***	1.03
	Hispanic	0.89	1.21*
	Non-Hispanic Asian	0.62***	0.95
	Non-Hispanic other	0.88	0.83
	Constant	0.027***	0.088***
Count model	Mode to work		
	Private vehicle	(*ref*)	–
	Public transit	1.09*	–
	Walk	1.68***	–
	Bike	1.27**	–
	Population density	1.01***	1.01**
	Percent rented	1.002**	1.004***
	Age	–	1.01**
	Age squared	–	0.9999**
	Constant	0.78**	0.79*
Wald chi-squared (*df*)		854.05*** (68)	646.43*** (67)
McFadden pseudo *R*^2^ (adjusted)		0.15	0.12

***p < 0.01.

**p < 0.05.

*p < 0.10.

*^a^Adjusted for education, whether the respondent has a medical condition that limits travel, whether a proxy respondent was used, number of trips taken on travel day, season of travel day, day of week of travel day, presence of heavy rail in metropolitan statistical area, and state fixed effects in both stages (logistic and count model)*.

Similarly, individuals who bike or take public transit to work are significantly more likely to be in the “not always zero” daily bike trip count group, compared to those who drive to work (OR = 300 and 2.99, respectively) (Table 2, logistic model). Increased population density is significantly associated with increased odds of taking at least one bike trip for working adults but not for non-working adults. Among individuals who take at least one bike trip per day, bicycle commuters take 1.48 times as many bike trips as those commuting by car (Table 2, count model).

**Table 2:** **Model for estimating daily number of bike trips**.

Variable		Odds ratio	
		Working adults^a^	Non-working adults^a^
Logistic model (probability not always zero)	Mode to work		
	Private vehicle	(*ref*)	–
	Public transit	2.99***	–
	Walk	1.31	–
	Bike	300***	–
	Population density	1.04*	–
	Age	–	0.98***
	Sex (*ref: male*)	0.29***	0.23***
	Race/ethnicity		
	Non-Hispanic White	(*ref*)	(*ref*)
	Non-Hispanic Black	0.61	0.52**
	Hispanic	0.88	0.49**
	Non-Hispanic Asian	0.43**	0.50
	Non-Hispanic other	0.49*	0.56
	Constant	0.0039***	0.059***
Count model	Mode to work		
	Private vehicle	(*ref*)	–
	Public transit	1.20	–
	Walk	0.91	–
	Bike	1.48***	–
	Sex (*ref: male*)	–	0.73**
	Race/ethnicity		
	Non-Hispanic White	(*ref*)	(*ref*)
	Non-Hispanic Black	1.22	1.28
	Hispanic	1.02	0.65
	Non-Hispanic Asian	1.36*	1.55***
	Non-Hispanic other	1.06	0.67
	Constant	1.51***	3.03*
Wald chi-squared (*df*)		79.5*** (28)	91.7*** (26)
McFadden pseudo *R*^2^ (adjusted)		0.29	0.12

***p < 0.01.

**p < 0.05.

*p < 0.10.

*^a^Adjusted for education, whether the respondent has a medical condition that limits travel, whether a proxy respondent was used, number of trips taken on travel day, season of travel day, day of week of travel day, presence of heavy rail in metropolitan statistical area, and census division fixed effects in both stages (logistic and count model)*.

Individual characteristics (age, sex, and race/ethnicity) have mixed associations in both the logistic and count portions of the models. Among employed adults, non-Hispanic Blacks and non-Hispanic Asians are less likely to be in the “not always zero” daily bike trip count group (OR = 0.64 and 0.62, respectively). Non-Hispanic Asian individuals are also less likely to be in the “not always zero” daily bike trip count group (OR = 0.43); however, those who are in the “not always zero” daily bike trip count group take 1.36 times more bike trips than non-Hispanic Whites (Table 2, count model). While gender has no significant effect on walking, men are much more likely to report biking for transportation, regardless of employment status.

### Walking and Biking Trip Purposes

To test the influence of explanatory variables on the distribution of walking and biking trip purposes, we fitted multinomial logistic regression models to NHTS data. Relative to a working adult who walks to work, a walk trip taken by an individual who commutes using a private vehicle, public transit, or bike is significantly more likely to be for a non-work purpose (shopping, social, recreational, or other purposes) (Table 3, top portion). For working adults, increased population density is associated with reduced odds that a given walk trip will be for recreation, and increased percentage of housing units that are rented is associated with increased odds that a given walk trip will be for shopping. For non-working adults, increased percentage of rental units is associated with increased odds that a given trip will be for non-recreational purposes (shopping, social, or personal/family business) (Table 3, bottom potion).

**Table 3:** **Model for estimating walk trip purpose**.

SUB-GROUP: WORKING ADULTS^a^				

Variable	Odds ratio for trip purpose (*base outcome: work trip*)			
	Shopping	Social	Recreational	Personal/family business
Mode to work				
Private vehicle	22.7***	35.2***	84.0***	28.1***
Public transit	11.3***	11.9***	12.8***	10.4***
Walk	(*ref*)	(*ref*)	(*ref*)	(*ref*)
Bike	19.5***	26.0***	25.1***	13.1***
Population density	1.002	1.02	0.965***	0.992
Percent rent	1.009**	0.998	1.00	1.00
Age	1.003	0.985**	1.01*	0.996
Race/ethnicity				
Non-Hispanic White	(*ref*)	(*ref*)	(*ref*)	(*ref*)
Non-Hispanic Black	1.12	0.587	0.427**	0.477***
Hispanic	1.04	0.790	0.914	0.752
Non-Hispanic Asian	0.745	0.360***	0.732	0.457**
Non-Hispanic other	0.718	0.713	0.929	0.570
Constant	0.038***	0.049***	0.035***	0.111***
Wald chi-squared (*df*)	1,610*** (124)		McFadden *R*^2^ (adjusted) 0.15	

**SUB-GROUP: NON-WORKING ADULTS^a^**				

**Variable**	**Odds ratio for trip purpose (*base outcome: recreational trip*)**			
	**Shopping**		**Social**	**Personal/family business**

Percent Rental	1.02***		1.02***	1.02***
Age	0.994*		0.984***	0.984***
Sex (*ref: male*)	1.10		1.01	1.30*
Race/ethnicity				
Non-Hispanic White	(*ref*)		(*ref*)	(*ref*)
Non-Hispanic Black	3.32***		1.72**	1.36
Hispanic	1.37		1.00	1.00
Non-Hispanic Asian	0.637		0.403**	0.895
Non-Hispanic other	1.30		0.687	0.842
Constant	0.291***		0.712	0.404**
Wald chi-squared (*df*)	525.7*** (84)		McFadden *R*^2^ (adjusted) 0.09	

***p < 0.01.

**p < 0.05.

*p < 0.10.

*^a^Adjusted for education, whether the respondent has a medical condition that limits travel, whether a proxy respondent was used, number of trips taken on travel day, season of travel day, day of week of travel day, presence of heavy rail in metropolitan statistical area, and census division fixed effects*.

Relative to a working adult who bikes to work, a bike trip taken by an individual using another commute mode is significantly more likely to be for a non-work purpose (shopping, social, recreational, or personal/family business) with two exceptions: no significant difference is found for the likelihood that a transit commuter takes a social bike trip or for the likelihood that someone who walks to work takes a personal/family business bike trip (Table 4, top portion). For working adults, built environment variables have no significant effects on bike trip purpose probabilities, while individual characteristics have mixed effects. For non-working adults, the proportion of trips that are for shopping increases significantly with population density, while the proportion of trips for business increases with percentage of rental units (Table 4, bottom portion).

**Table 4:** **Model for estimating bike trip purpose**.

SUB-GROUP: WORKING ADULTS^a^				

Variable	Odds ratio for trip purpose (*base outcome: work trip*)			
	Shopping	Social	Recreational	Personal/Family Business
Mode to work				
Private vehicle	21.0***	18.5***	165***	28.9***
Public transit	7.81***	0.908	8.71***	6.23**
Walk	10.0**	15.3***	20.6***	6.60
Bike	(*ref*)	(*ref*)	(*ref*)	(*ref*)
Age	0.934	0.924	0.902	0.806***
Age squared	1.001	1.001	1.002**	1.002***
Race/ethnicity				
Non-Hispanic White	(*ref*)	(*ref*)	(*ref*)	(*ref*)
Non-Hispanic Black	3.35*	0.529	2.39	1.70
Hispanic	5.41***	1.93	3.18**	1.10
Non-Hispanic Asian	4.48	0.143	2.51	1.90
Non-Hispanic other	1.05	5.30	4.33	4.09
Constant	0.0044***	0.080	0.0023***	2.19
Wald chi-squared (*df*)	503.3*** (*100*)		McFadden *R*^2^ (adjusted): 0.38	

**SUB-GROUP: NON-WORKING ADULTS^a^**				

**Variable**	**Odds ratio for trip purpose (*base outcome: recreational trip*)**			
	**Shopping**		**Social**	**Personal/family business**

Population density	1.11**		1.04	0.993
Percent rental	1.01		1.01	1.02**
Age	1.03***		0.995	0.972***
Race/ethnicity				
Non-Hispanic White	(*ref*)		(*ref*)	(*ref*)
Non-Hispanic Black	1.86		1.67	0.77
Hispanic	0.518		0.571	0.21*
Non-Hispanic Asian	6.65**		2.25	8.01**
Non-Hispanic other	0.0304**		0.669	0.0622***
Constant	0.114**		1.49	4.09
Wald chi-squared (*df*)	327.7*** (84)		McFadden *R*^2^ (adjusted) 0.28	

***p < 0.01.

**p < 0.05.

*p < 0.10.

*^a^Adjusted for education, whether the respondent has a medical condition that limits travel, whether a proxy respondent was used, number of trips taken on travel day, season of travel day, day of week of travel day, presence of heavy rail in metropolitan statistical area, and Census region fixed effects*.

*^b^Same adjusted as above, with the exception of census division fixed effects in place of Census region fixed effects*.

### Duration of Walking and Biking Trips

To test the influence of commute mode to work, individual characteristics, and built environment variables on trip durations, we fit GEE models predicting trip duration to the NHTS data. Relative to a walk trip to work by someone who typically walks to work, all other walk trips are longer with the exception of walk trips to work by individuals who typically commute via transit or private vehicle (Table 5). Thus, walk trips for purposes other than commuting to work are typically longer than walks to work. Additionally, the significantly shorter walk trips to work for those typically commuting via transit likely reflect walking shorter distances to and/or from transit stops at the beginning and/or end of work commutes. Travel time to work is intuitively associated with the duration of walking trips to work; much smaller but significant associations with other trip types may reflect an unobserved non-aversion for longer trip durations. For non-working adults with no commute to work, shopping, social, and personal/family business walk trips are significantly shorter than recreational trips. Older individuals take longer walk trips, perhaps reflecting decreased walking speed. Additionally, Hispanic and non-Hispanic Blacks take significantly longer walk trips than non-Hispanic White individuals.

**Table 5:** **Model for estimating walk trip duration**.

Variable	Regression coefficient	
	Working adults^a^	Non-working adults^a^
Trip purpose	
Shopping trip	–	−0.711***
Social trip	–	−0.763***
Recreational trip	–	(*ref*)
Personal/family business trip	–	–0.459***
Interaction: trip purpose with mode to work		
Work trip × private vehicle to work	0.043	–
Work trip × transit to work	−0.404***	–
Work trip × walk to work	(*ref*)	–
Work trip × bike to work	0.388***	–
Shopping trip × private vehicle to work	1.02***	–
Shopping trip × transit to work	1.12***	–
Shopping trip × walk to work	1.16***	–
Shopping trip × bike to work	1.26***	–
Social trip × private vehicle	1.07***	–
Social trip × transit to work	1.03***	–
Social trip × walk to work	1.25***	–
Social trip × bike to work	1.28***	–
Recreational trip × private vehicle to work	2.08***	–
Recreational trip × transit to work	2.05***	–
Recreational trip × walk to work	2.13***	–
Recreational trip × bike to work	2.13***	–
Personal/family business trip × private vehicle	1.30***	
Personal/family business trip × transit to work	1.21***	–
Personal/family business trip × walk to work	1.29***	–
Personal/family business trip × Bike to work	1.32***	–
Interaction: log of time to work with trip purpose		
Log time to work × work trip	0.537***	–
Log time to work × shopping trip	0.063**	–
Log time to work × social trip	0.080***	–
Log time to work × recreational trip	−0.020**	–
Log time to work × personal/family business	0.070***	–
Interaction: population density with trip purpose		
Population density × work trip	0.004*	–
Population density × shopping trip	−0.003	0.001
Population density × social trip	0.008**	0.011***
Population density × recreational trip	−0.004**	−0.003
Population density × personal/family business	−0.001	0.002
Interaction: percent rental units with trip purpose		
Percent rental × work trip	0.002***	–
Percent rental × shopping trip	0.002**	0.003***
Percent rental × social trip	−0.0003	0.001
Percent rental × recreational trip	−0.001*	−0.001
Percent rental × personal/family business trip	−0.0001	−0.0001
Age	0.002***	0.006***
Age squared	–	−0.0001***
Sex (*ref: male*)	–	−0.083***
Race/ethnicity		
Non-Hispanic White	(*ref*)	(*ref*)
Non-Hispanic Black	0.084***	0.103***
Hispanic	0.121***	0.136***
Non-Hispanic Asian	0.008	0.036
Non-Hispanic other	0.006	0.053
Constant	0.94***	3.20***
Wald chi-squared (*df*)	4,841*** (*102*)	2,680*** (*81*)

***p < 0.01.

**p < 0.05.

*p < 0.10.

*^a^Adjusted for education, whether the respondent has a medical condition that limits travel, whether a proxy respondent was used, number of trips taken on travel day, season of travel day, day of week of travel day, presence of heavy rail in metropolitan statistical area, and state fixed effects*.

Somewhat paradoxically, increased population density and percent rental units are associated with slightly longer walk trips to work. Increased population density is also associated with slightly longer walking trips for social purposes, and increased percent rental units is associated with slightly longer shopping trips. While increases in these built environment variables would seemingly be associated with an increased density of destinations and thereby shorter trip distances, these built environment variables also may be associated with increased replacement of slightly longer duration non-walking trips with walking trips, thus increasing average trip duration. Increased population density and percent rental units are both associated with shorter recreational walking trips, possibly because recreational destinations are closer to residential areas.

Similar associations between trip duration, trip purpose, and built environment variables occur for biking trips (Table 6). Some differences exist regarding associations with trip type and mode to work: relative to a bike trip to work by someone who typically cycles to work, a work bike trip by someone who typically drives to work is significantly longer. Bike trips to work by someone who typically walks to work are shorter than those taken by someone who typically bikes to work. Finally, work bike trip duration is not significantly associated with taking public transit to work, likely reflecting the relative rarity of bike trips to access public transit. While population density not associated with bike trip durations, percentage of rental units is negatively associated with the duration of shopping and recreational bike trips for working adults. For non-working adults, shopping, social, and personal/family business bike trips are significantly shorter than the reference category (recreational trips). Among working adults, age exhibits a significant quadratic relationship with bike trip duration. Among working and non-working adults, women take shorter bike trips compared to men.

**Table 6:** **Model for estimating bike trip duration**.

Variable	Regression coefficient	
	Working adults^a^	Non-working adults^a^
Trip purpose		
Shopping trip	–	−0.579***
Social trip	–	−0.449***
Recreational trip	–	(*ref*)
Personal/family business trip	–	−0.388***
Interaction: Trip purpose with mode to work		
Work trip × private vehicle to work	0.378***	–
Work trip × transit to work	0.015	–
Work trip × walk to work	−0.196**	–
Work trip × bike to work	(*ref*)	–
Shopping trip × private vehicle to work	0.987**	–
Shopping trip × transit to work	0.900*	–
Shopping trip × walk to work	0.954**	–
Shopping trip × bike to work	0.970***	–
Social trip × private vehicle	1.59***	–
Social trip × transit to work	1.38***	–
Social trip × walk to work	1.90***	–
Social trip × bike to work	1.58***	–
Recreational trip × private vehicle to work	2.44***	–
Recreational trip × transit to work	2.29***	–
Recreational trip × walk to work	2.75***	–
Recreational trip × bike to work	2.53***	–
Personal/family business trip × private vehicle	1.31***	–
Personal/family business trip × transit to work	0.939**	–
Personal/family business trip × walk to work	1.09***	–
Personal/family business trip × bike to work	1.19***	–
Interaction: log of time to work with trip purpose		
Log time to work × work trip	0.731***	–
Log time to work × shopping trip	0.358***	–
Log time to work × social trip	0.178*	–
Log time to work × recreational trip	0.0460	–
Log time to work × personal/family business	0.297***	–
Interaction: percent rental units with trip purpose		
Percent rental × work trip	−0.0004	–
Percent rental × shopping trip	−0.005***	–
Percent rental × social trip	−0.003	–
Percent rental × recreational trip	−0.004***	–
Percent rental × personal/family business trip	−0.002	–
Age	0.019**	–
Age squared	−0.0002*	–
Sex (*ref: male*)	−0.075*	−0.190***
Race/ethnicity		
Non-Hispanic White	–	(*ref*)
Non-Hispanic Black	–	0.404***
Hispanic	–	0.191*
Non-Hispanic Asian	–	0.280
Non-Hispanic other	–	0.062
Constant	0.46*	3.33***
Wald chi-squared (*df*)	1,085*** (*53*)	168.8*** (29)

***p < 0.01.

**p < 0.05.

*p < 0.10.

*^a^Adjusted for education, whether the respondent has a medical condition that limits travel, whether a proxy respondent was used, number of trips taken on travel day, season of travel day, day of week of travel day, presence of heavy rail in metropolitan statistical area, and census division fixed effects*.

**Figure 2:**
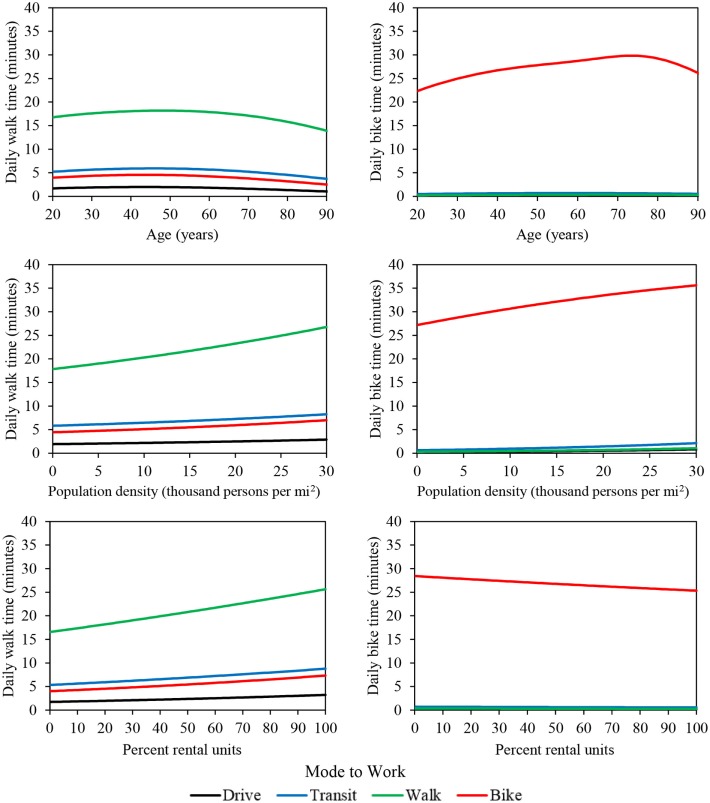
**Regression estimates of daily walking and biking time as a function of age, population density, and percent rental units**. In each plot, median values are used for all other variables.

To illustrate the combined effects of the models summarized in Tables 1–6, Figure 2 presents estimates of weekday walking and biking time for a median individual in each commuter category. Generally, individuals who walk to work have much higher average daily walking time than other types of commuters. Similarly, bicycle commuters have higher average daily biking time than all other commuters. Transit commuters have moderate daily average walking times, likely reflecting walk trips to and from transit stops. Bike commuters also have moderate daily average walking times. Daily walking time for individuals who walk to work peaks around age 50 and then decreases slightly with age, while daily biking time peaks at a later age for bicycle commuters. Increases in daily bike time for bike commuters until to around age 75 is a surprising finding, perhaps reflecting strong underlying preferences for biking among those that continue to bike to work at older ages. Both daily walking and biking time increase as population density and percent rental units increase.

### Effects of Commuting Method and Built Environment Variables on Physical Activity

**Figure 3:**
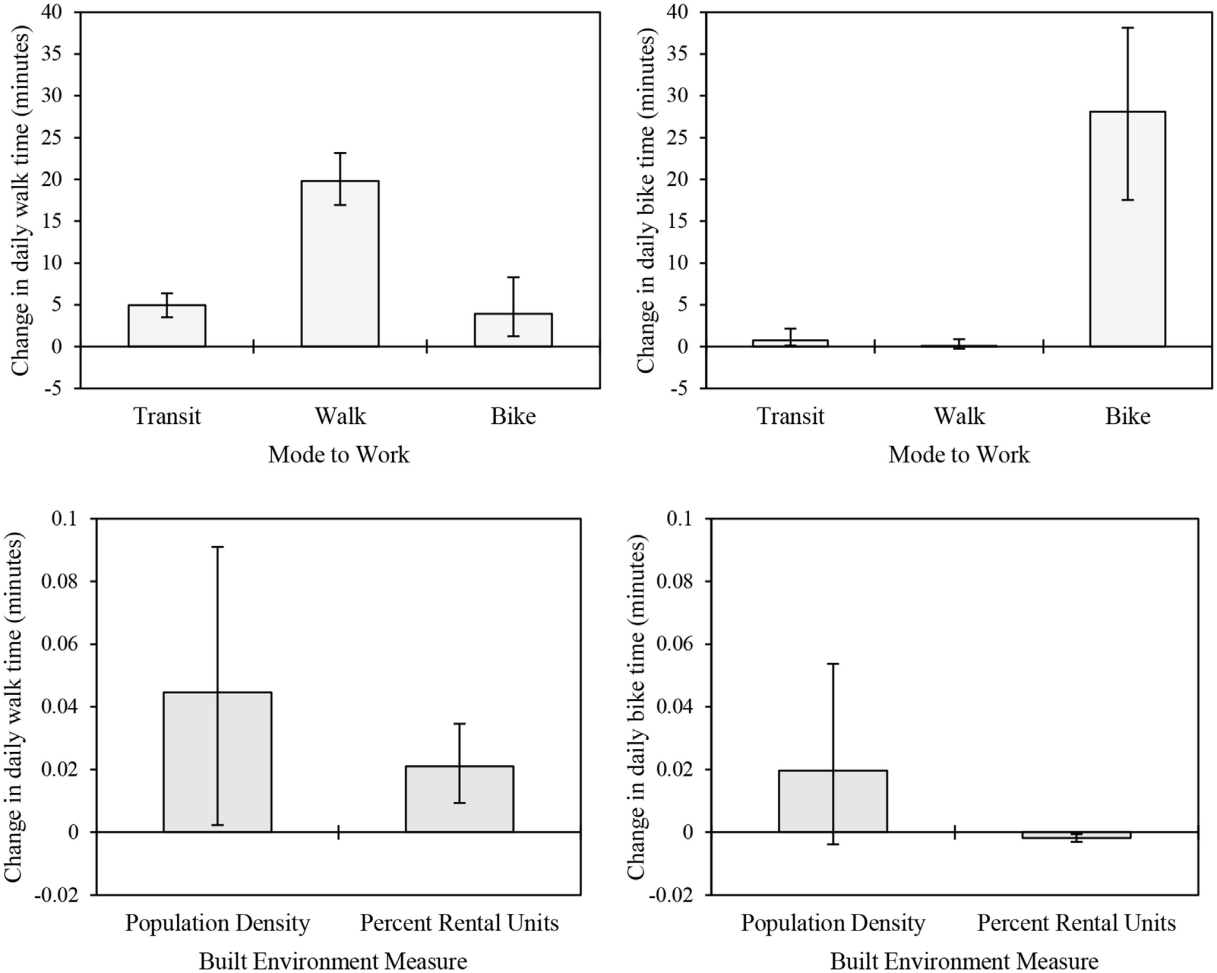
**Effects of commuting method on daily time spent walking (top left) and biking (top right) relative to the reference category (driving a private vehicle to work), and effects of 1-unit changes in built environment measures on daily walking (bottom left) and biking (bottom right) time**.

To demonstrate the effect of commuting method, population density, and percent rental units on physical activity, we calculated the average marginal effects of a 1-unit change in each of these variables on daily walking and biking times. Average marginal effects for commute mode represent the average increase in daily walking or biking time expected given a switch from the reference category (private vehicle) to a different commuting mode. Average marginal effects for population density and percent rental units both represent the average change in daily walking or biking time given a one unit change in these variables. On average, an individual who walks to work walks an additional 19.8 (95% CI 16.9–23.1) minutes per day compared to an individual who drives to work. Transit and bicycle commuters walk an additional 5.0 (95% CI 3.5–6.4) and 3.9 (95% CI 1.2–8.3) minutes per day, respectively, compared to drivers (Figure 3; top left). The effect of biking to work on daily biking time is stronger than the effect of walking to work on daily walking time: a bicycle commuter bikes an additional 28.0 (95% CI 17.5–38.1) minutes per day compared to drivers. Transit commuters cycle for an additional 0.8 (95% CI 0.1–2.2) minutes per day compared to drivers (Figure 3; top right). However, individuals who walk to work do not bike significantly more than drivers. Built environment variables have small but significant effects on daily walking time but no significant effects on daily biking time. For working adults, a 1-Unit increase in population density (thousands of people per square mile) increases daily walking time by 0.05 (95% CI 0.002–0.1) minutes, and a 1-unit increase in percent rental units increases daily walking time by 0.02 (95% CI 0.01–0.04) minutes.

Average marginal effects for individual models (trip count, purpose, and duration) and are presented in the Supplementary Material. Active commuters generally take significantly more walk and/or bike trips per week, but these trips tend to have shorter durations. Thus, the net effect of commute mode to work on weekly walking or biking time (Figure 3) is slightly less than the effect of commute mode on the number of weekly walking or biking trips (Table S5 in Supplementary Material). For example, a non-Hispanic White individual who walks to work is expected to take 1.6 (1.4–1.7) additional walk trips per day relative to a similar individual who drives to work (Figure S3 in Supplementary Material). For this same individual, the likelihood that a given walk trip would be for work purposes is 38% (33–43%) greater than their counterpart who drives to work (Figure S4 in Supplementary Material). Finally, for this individual, a typical work trip would have a duration 5.2 (3.0–7.5) minutes shorter than a recreational trip (Figure S5 in Supplementary Material). Thus, while active commuters take a much greater number of walk or bike trips per day, it is more likely that trips taken by active commuters will have shorter durations than trips taken by individuals who drive to work due to the shift toward work-related active travel. This nuance highlights the importance of including trip probability models in the initial estimation framework presented in Eq. 1.

### Model Validation

To assess the regression models’ accuracy, we used the models and Eqs 1–2 to estimate daily physical activity from walking and biking for all participants in the 2006 Greater Triangle Travel Survey (23), and we compared the estimates to the survey results. The models estimate an average of 0.22 MET-hours per day of walking and biking for those who drive to work; the averaged observed value for private vehicle commuters is 0.20 MET-hours per day. For transit commuters, the models estimate an average of 0.78 MET-hours per day compared to an average observed value of 1.44 MET-hours per day. For those who walk to work, the models predict an average of 1.46 MET-hours per day, compared to an average observed value of 1.54 MET-hours per day. Finally, for bike commuters, the model estimates is 3.96 MET-hours per day compared to an average observed value of 5.23 MET-hours.

**Figure 4:**
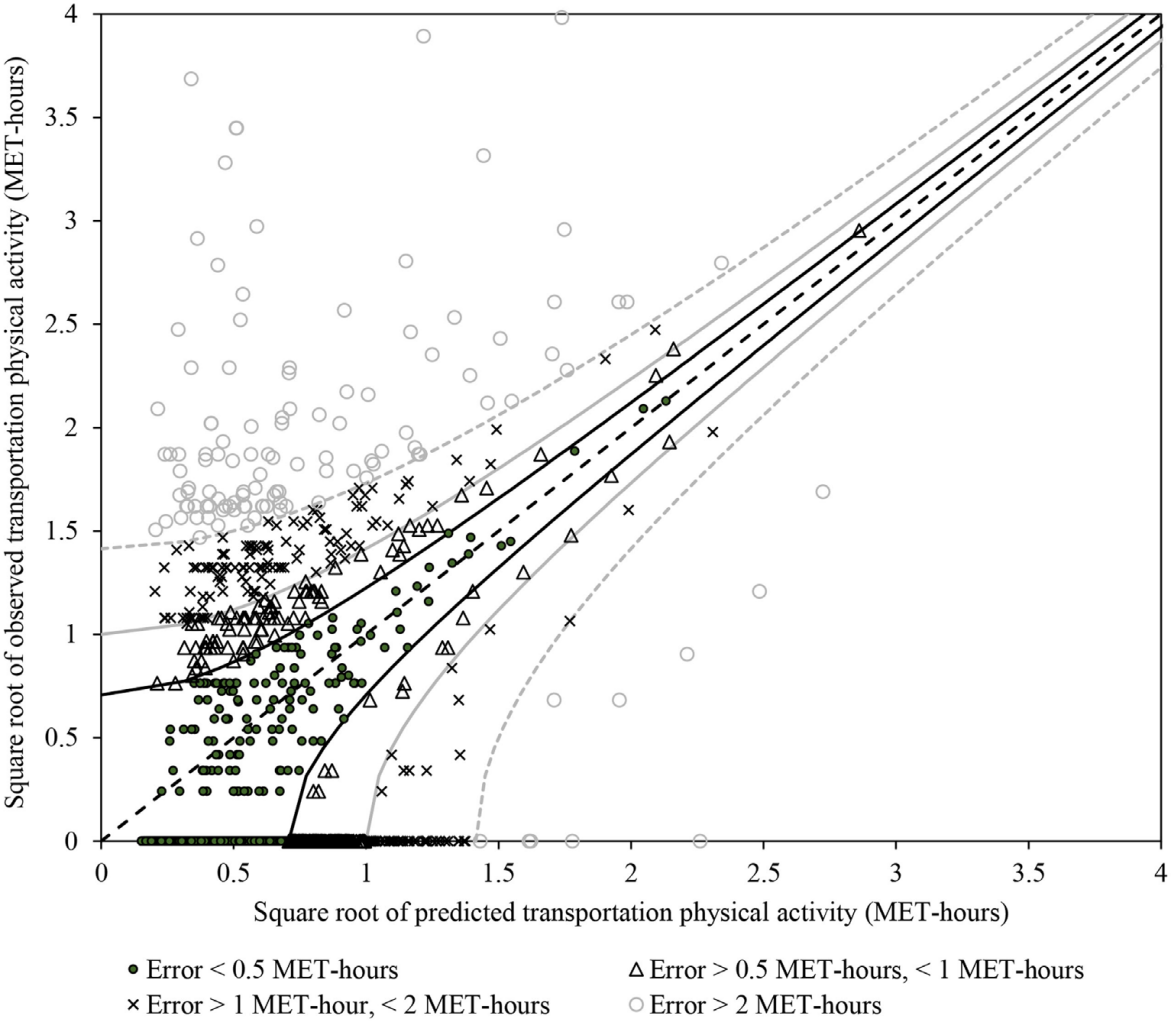
**Predicted versus observed transportation physical activity for the validation dataset**. Dashed black line: perfect agreement. Solid black lines and circular markers: predictions within 0.5 MET-hours per day of observed values. Solid gray lines and triangular markers: predictions within 1 MET-hour per day of observed values. Dashed gray lines and x-shaped markers: predictions within 2 MET-hours per day of observed values. Hollow circle markers: predictions more than 2 MET-hours different than observed values.

The square root of model predictions are plotted against the square root of observed values in Figure 4 along with lines representing perfect agreement (dashed black line) and predictions within 0.5 (solid black lines), 1 (solid gray lines), and 2 (dashed gray lines) MET-hours per day. Solid black circles, black triangles, gray crosses, and gray circles represent individual estimates within 0.5, 1, 2, or more than 2 MET-hours per day, respectively. Estimated physical activity from walking and biking is within 0.5, 1, and 1.6 MET-hours per day for 83, 91, and 95% of observations, respectively. The Triangle Travel Survey contains a large proportion of days with no walking or biking trips, which are clustered along the *x*-axis. While the NHTS model estimates non-zero transportation physical activity for these days, predictions are <0.2 MET-hours per day for 63% of observed zeroes and <0.62 MET-hours per day for 95% of observed zeroes.

Overall, the NHTS model performs very well for those who walk or drive to work. However, the model under-estimates physical activity for those who bike or ride transit to work. Under-predictions for transit use may reflect inclusion of more individuals using park-and-ride lots to access transit services in the NHTS dataset than in the Raleigh–Durham–Chapel Hill region, where park-and-ride lots are available only for regional bus service. Under-estimates of physical activity for bicycle commuters may reflect the limited availability of travel time to work information for cyclists in the Triangle Travel Survey.

### Health Impacts of Active Transportation in the Case Study Region

**Figure 5:**
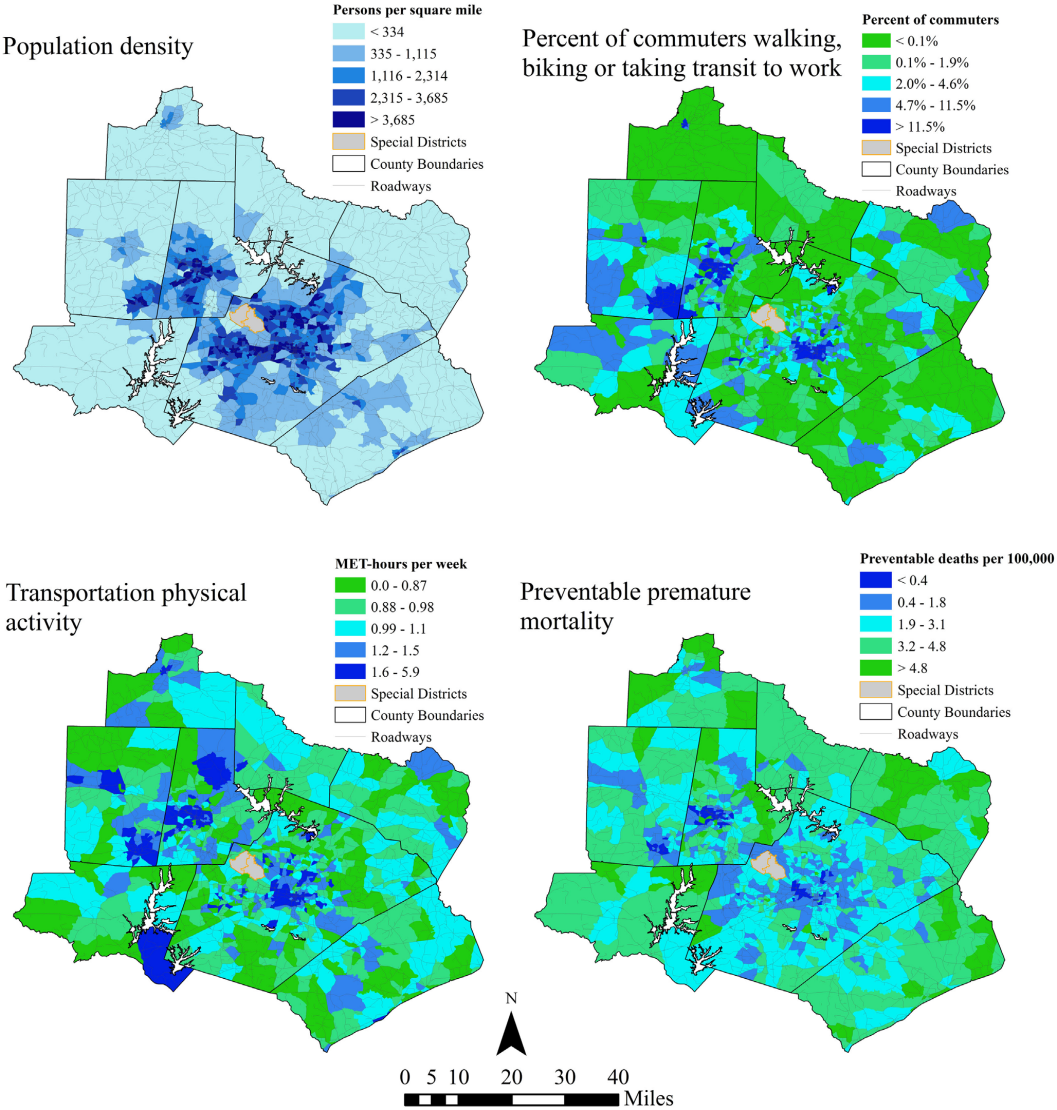
**Study region population density (top left), proportion of commuters walking or biking to work (top right), estimated weekly transportation physical activity (bottom left), and preventable mortality per 100,000 people in 2013**. Special districts indicated in the maps include an international airport and a state park.

Using Eqs 1–2, the population-weighted mean transportation physical activity level for the Raleigh–Durham–Chapel Hill region is 1.2 MET-hours per week. Generally, block groups with high population density (Figure 5, top left panel) and/or high proportions of the population who walk or bike to work (Figure 5, top right panel) tend to also have higher estimated transportation physical activity generally. Averaging estimated transportation physical activity within population density quintiles of block groups confirms this observation: the bottom two quintiles have similar average estimated transportation physical activity while estimated transportation physical activity increases incrementally in the top three quintiles (Table 7). Average estimated transportation physical activity in the highest quintile of population is 81% greater than average estimated transportation physical activity in the lowest quintile (Table 7).

**Table 7:** **Effects of population density on transportation physical activity and estimates of preventable premature deaths relative to the walkable neighborhoods counterfactual**.

Quintile of population density (persons/mi^2^)	Mean population density (persons/mi^2^)	Population	Transportation physical activity (MET-h/week)	Preventable mortality (deaths per 100,000)	Preventable mortality (total deaths)
1	165.4	314,734	1.00	3.6	11
2	688.4	369,457	1.01	2.7	9.8
3	1,711	327,809	1.16	2.3	7.7
4	2,913	341,956	1.33	1.8	6.2
5	5,954	311,268	1.81	0.93	2.9
All	2,165	1,656,225	1.20	2.3 (0.88–3.6)	38 (15–59)

Estimated transportation physical activity levels were used to estimate the number of premature deaths that could be prevented if all individuals walked 34.7 min per week, as observed in walkable neighborhoods in Baltimore and Seattle (26). According to this estimate, 38 (95% CI 15–59) additional premature deaths would have been avoided across the region As shown in Figure 5 (bottom right panel), the health risks posed by low transportation physical activity, relative to expected transportation physical activity for walkable neighborhoods, are lowest in block groups with high population density and/or high proportions of the population walking or biking to work. As expected, the spatial pattern of estimated health impacts is roughly the inverse of the spatial pattern of transportation physical activity. Premature mortality that could be avoided if all individuals in the study region walked 34.7 min per week decreases with population density, suggesting that population density supports transportation physical activity and reduces health risks associated with low physical activity (Table 7). Equivalently, prevented premature mortality is nearly four times greater in the highest population density quintile compared to the lowest.

### Hypothetical HIA Application

To demonstrate how our regression models could be used to support active transportation HIA, we developed three hypothetical scenarios in which changes made to the built environment increase transportation physical activity in the Raleigh–Durham–Chapel Hill region. In the first, transportation physical activity is assumed to increase by 7.9% for all individuals in the study region as a result of 10% increases in land-use diversity, transit stop coverage, and intersection density. In the second, 7.9% of drivers begin walking to work, increasing population-average transportation physical activity by 0.34 MET-hours per week. In the third, 14.5% of drivers switch to commuting by public transit, increasing average transportation physical activity by 0.24 MET-hours per week (Table 8). Compared to baseline conditions, these three scenarios would reduce premature mortality across the region by 3.2 (95% CI 1.3–5.2), 8.0 (95% CI 3.2–12.5), and 6.2 (95% CI 2.6–10.3) deaths per year, respectively. While only illustrative, the application of our regression models to predict health benefits of hypothetical changes in the built environment demonstrates how such models could be used to support quantitative HIAs of built environment changes that support walking and biking for transportation. The first scenario illustrates how our regression models could support the calculation of population-wide increases in physical activity while the second and third scenario illustrate how these models could instead support HIAs of built environment changes that result in shifts of transportation mode used for the work commute.

**Table 8:** **Transportation physical activity and health benefits estimated for hypothetical built environment changes**.

	Scenario 1: population increase in walking	Scenario 2: drivers shift to walking	Scenario 3: drivers shift to transit
Transportation physical activity (MET-h/week)	1.32	1.56	1.47
Increase in transportation physical activity, relative to baseline (MET-h/week)	0.10	0.34	0.24
Prevented mortality (total deaths)	3.2 (1.3–5.2)	8.0 (3.2–12.5)	6.2 (2.6–10.3)
Prevented mortality (deaths per 100,000)	0.20 (0.08–0.31)	0.96 (0.38–1.5)	0.70 (0.39–1.2)

## Discussion

### Overall Significance

Using data from the 2009 NHTS, we developed regression models that future analysts can use to predict weekly time spent walking and biking for transportation based on routinely collected demographic and built environment data. These models enabled the development of transportation physical activity predictions across the Raleigh-Durham-Chapel Hill case study region with greater spatial resolution than was previously possible. We showed how the models can be used to estimate the potential health benefits of increasing walking and biking in the case study region: for example, if changes to the built environment induced 14.5% of drivers to commute by public transit, an estimated 6.2 (95% CI 2.6–10.3) premature deaths could have been prevented in 2013. Further, estimates of health impacts for baseline transportation physical activity at the Census block groups scale across the region (Figure 5) could be used to target built environment changes to better support walking and biking for transportation. Physical activity estimates at this fine scale of geographic resolution enable better understanding of how risks associated with physical inactivity vary across urban areas. As transportation HIA continues to evolve, more advanced modeling techniques are emerging. While advanced modeling tools offer a number of benefits to transportation HIA, they may have extensive data requirements (32). The estimation approach presented in this paper provides a means to estimate baseline transportation physical activity levels and compare baseline levels across space using readily accessible data.

More broadly, a handful of recent studies have explored the competing health risks posed by transportation systems in urban environments. While compact urban environments support increased walking and biking for transportation, residents of densely populated neighborhoods may be exposed to more air pollution (35, 36). Additionally, active commuters may have increased exposure relative to non-active commuters due to increased inhalation rates (37). However, estimates suggest that the benefits of transportation physical activity for active commuters outweigh risks associated with increased air pollution exposure (38). A previous study in the Raleigh–Durham–Chapel Hill metropolitan area estimated that, in 2010, 47 premature deaths were associated with exposure to fine particulate matter air pollution from motor vehicles (36). Other recent work provides evidence that residents in denser neighborhoods may face greater health risks from exposure to pollutants in ambient air (35). Thus, physical activity and air pollution exposure may respond to characteristics of the built environment in different directions and with different magnitudes. While a variety of tools and methods exist to estimate air pollution exposures at fine spatial resolutions (36, 39, 40), this study presents a novel estimation framework for estimating active transportation behaviors at fine spatial resolutions across a large metropolitan region. In doing so, we support future research efforts to identify the relationships between the built environment and competing transportation health risks in urban areas. Across urban areas, these competing risks result in a highly heterogeneous riskscape. Quantitative assessments of these risks support informed policy-making to reduce the health risk associated with transportation.

### Comparison to Previous Studies

Previous analyses of the NHTS have found a number of associations between individual characteristics and active transportation behaviors. For example, Pucher et al. found that men are much more likely to cycle at least 30 min per day while women are slightly more likely to walk at least 30 min per day (19). Similarly, we find that men are much more likely to take at least one bike trip compared to women (Table 2). In contrast to previous work finding that individuals who ride public transit walk 21 min per day, we find that individuals who take transit to work walk an additional 4.5 min per day compared to individuals who commute using a private vehicle (6). This discrepancy may arise for several reasons. First, our estimate includes individuals who use all forms of public transit, including paratransit services. Since commuters do not have to walk or bike to access demand-responsive services, the average marginal effect of taking public transit to work is attenuated. Second, we include transit commuters who do not walk or bike to access transit (e.g., park-and-ride users). Third, we calculated the marginal effect of riding transit to work relative to driving. Individuals who drive to work still walk and bike for other purposes, and our results show that taking public transit increases the likelihood that a given trip will be for work purposes (Table 3). Thus, we estimate the impact of transit commuting to a non-zero baseline and find some evidence that transit users shift the purpose of walk trips toward commuting and away from other purposes. Previous work has also found that individuals who walk to public transportation are more likely to be non-White (6). Counter to this finding, we find that non-Hispanic Blacks and Asians are less likely to take at least one walking trip in a given day (Table 1). However, we also find that non-Hispanic Blacks take longer walk trips, counteracting the effect of lower trip counts on daily walking time (Table 5). These differences are likely due to our use of commute mode to work as an explanatory variable. Non-White individuals are more likely to ride transit to work; thus, the correlation between race/ethnicity and commute mode to work may attenuate the relationship between race/ethnicity and daily walking trips.

Assessing active transportation behaviors at the neighborhood scale, a number of previous studies have shown that individuals living in more walkable neighborhoods are more physically active than residents in non-walkable neighborhoods (10, 26, 33, 34). Broadly, our findings are aligned with these previous neighborhood-scale studies. We found strong effects of commute mode choice on daily walking and biking time, as well as small yet significant associations between built environment measures and daily walking time (Figure 3). Overall, we found the highest population-average levels of physical activity – and, in turn, the lowest burden of preventable premature mortality associated with physical inactivity – in the densest quintile of block groups in the region (Table 7). Thus, our regional analysis using a downscaled national survey largely aligns with previous studies conducted at the neighborhood scale.

### Limitations

This analysis considers only physical activity from transportation in estimating preventable mortality relative to counterfactual scenarios in which more people walk for transportation. Because the dose–response function linking transportation physical activity to all-cause mortality (Eq. 6) is log-linear, the slope of the function decrease as dose increases. Thus, estimated risk reduction for a fixed increase in physical activity is sensitive to the baseline level of physical activity. This may lead us to overestimate preventable mortality. However, the meta-analysis that derived Eq. 6 included studies that controlled for physical activity on other domains when estimating the dose–response function for transportation walking and biking (29). Thus, Eq. 6 implicitly assumes that there is some unobserved level of non-transportation physical activity in the population. While considering only transportation physical activity is a limitation of our approach, the tendency of this limitation to result in overestimation of preventable mortality is minimized by the use of a dose–response function that accounts for non-transportation physical activity.

Additionally, the 2009 NHTS offers only a snapshot of walking and biking behaviors across the US at a single point in time. The NHTS was previously administered in 2001. Comparisons of walking in biking in the 2001 and 2009 NHTS reveal several small, yet significant, trends in active transportation behaviors (19). However, the data are insufficient to project baseline trends or link these behaviors to exogenous variables. As population cohorts age and economic conditions (e.g., gasoline prices) change, preferences for active transportation may also change. However, our model validation shows that regression estimates from the NHTS have a reasonable predictive validity.

Finally, the generation of block group population distributions across individual-level dimensions assumes that the distributions of different population characteristics are independent when cross-tabulations were not available at the block group level in the ACS (e.g., the distribution of commute mode to work for working adults was assumed to be independent of the distribution of race). Finally, the ACS groups all public transit services into a single category when reporting commute mode to work at the block group geography, including demand-responsive paratransit services in rural areas. These transit services may not be associated with as much walking and biking for transportation as fixed-route transit service in urban areas. Thus, in some rural block groups, this may result in an overestimation of transportation physical activity. Despite limitations associated with the ACS data, our approach offers a much more detailed understanding of active transportation behaviors than is offered by existing routinely collected data sources.

## Conclusion

As understanding of the connections between the built environment and public health evolve, tools and methods to develop robust population-level estimates of physical activity from walking and biking must be developed alongside models to characterize exposure to other transportation health risks, such as air pollution. This study demonstrates a statistical approach to characterizing walking and biking levels across a large metropolitan area using routinely collected data. This approach is useful both for estimating baseline behaviors in support of transportation HIAs and for comparing the magnitude of risks associated with physical inactivity to other competing health risks in urban areas. In a case study application, we used this approach to highlight the potential health benefits of modifying the built environment to support walking, biking, and riding public transit to work. In future work, similar approaches could lead to more detailed understanding of how the design of urban environments affects multiple health risks, including physical inactivity, exposure to air pollution, and traffic accidents. Clarifying the complex interplay of competing health risks associated with transportation systems in urban areas is an important research direction to improve understanding of population-level health impacts of the built environment. Ultimately, tools to support quantitative HIAs can support more robust consideration of multiple health risks when deciding how to shape the built environment.

## Author Contributions

TM performed data analysis, regressions modeling, and health impact assessment. JG advised the development of regression models and supported the health impact assessment.

## Conflict of Interest Statement

The authors declare that the research was conducted in the absence of any commercial or financial relationships that could be construed as a potential conflict of interest.
