# Supplementary material for: Estimating Active Transportation Behaviors to Support Health Impact Assessment in the United States
Source: Front Public Health. 2016 May 2;4:63. doi: 10.3389/fpubh.2016.00063 (PMC4852202; doi:10.3389/fpubh.2016.00063)
Supplement: Supplementary file 1 [file data_sheet_1.docx]

# Step-by-step example of calculating health impact estimates

A step-by-step explanation of estimating transportation physical activity, assigning these estimates to a population distribution in a block group, and using these estimates to develop health impact estimates for an example block group is provided below. Block Group 2, Census Tract 107.03 in Orange County, North Carolina had 2,142 residents in 2013. This block group has a relatively high share of active commuters, with 70% of commuters traveling to work by car or working at home, 21% taking public transit to work, and 9% biking to work. The population is composed of 69% non-Hispanic White individuals, 9% non-Hispanic Black individuals, 12% Hispanic individuals, 3% non-Hispanic Asian individuals, and 7% non-Hispanic other individuals. This block group also has a high share of residential units that are rented (62.4%) and a higher than average population density (4,700 persons per square mile).

## Step 1: Estimating daily walk and bike trip counts for workers and non-workers

## Regression coefficients from the zero-inflated Poisson models are used to estimate daily walking and biking trips for a typical weekday and a typical weekend, once for workers and once for non-workers. Coefficients for explanatory variables are provided in Table 1 (walk trips) and Table 2 (bike trips). Coefficients for model controls are not presented in text but included in the application below. These models are estimated for all possible combinations of individual-level variables within each block group. Area-level variables (e.g., population density) vary between block groups; thus, all possible combinations of individual-level variables share the same area-level variables within a block group. This generates eight sets of estimates for $\boldsymbol{E}\left( \boldsymbol{t}_{\boldsymbol{m}\boldsymbol{,i}} \right)$ in Equation 1:

1. Typical weekday walk trips for working adults, $E\left( t_{m=walk,i} \right)|weekday, working$
2. Typical weekend walk trips for working adults, $E\left( t_{m=walk,i} \right)|weekend, working$
3. Typical weekday walk trips for non-working adults, $E\left( t_{m=walk,i} \right)|weekday, non working$
4. Typical weekend walk trips for non-working adults, $E\left( t_{m=walk,i} \right)|weekend, nonworking$
5. Typical weekday bike trips for working adults, $E\left( t_{m=bike,i} \right)|weekday, working$
6. Typical weekend bike trips for working adults, $E\left( t_{m=bike,i} \right)|weekdend, working$
7. Typical weekday bike trips for non-working adults, $E\left( t_{m=bike,i} \right)|weekday, nonworking$
8. Typical weekend bike trips for non-working adults, $E\left( t_{m=bike,i} \right)|weekend, nonworking$

Each of these sets of estimates contains unique values for each possible combination of age (ranging from 18-95), sex (male or female), race (non-Hispanic White, non-Hispanic Black, Hispanic, non-Hispanic Asian, or non-Hispanic other), and, for working adults, mode to work (drive, transit, walk, or bike). These estimates are stored in a matrix $\mathbf{TPA}_{\mathbf{count}}$ containing 3,900 cells (78 possible ages, two possible sexes, five possible races, and five possible modes to work, including non-working as a fifth mode). For our example block group, estimates of typical weekday daily walk trips for a working, non-Hispanic Black adult are provided below, by commute to work (Figure S7):


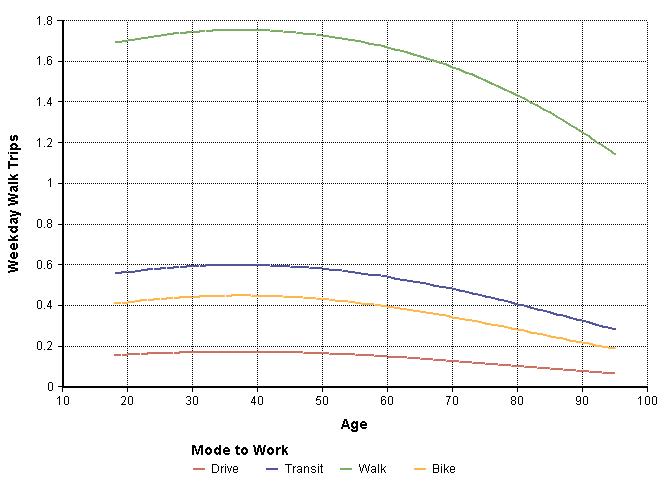


**Figure S7.** Predictions of typical weekday walk trips for a non-Hispanic Black working adult living in the example block group

## Step 2: Estimating walk and bike trip purpose probabilities for workers and non-workers

Regression coefficients from the multinomial logistic regression models are then used to estimate the probability that a given walk or bike trip for a specific individual is for one of the five purposes outlined in the text. Regression coefficients for these models appear in Table 3 (walk trips) and Table 4 (bike trips). Using the same dimensions as above, these models are used to estimate $\boldsymbol{Pr}\left( \boldsymbol{p}_{\boldsymbol{m}\boldsymbol{,i}} \right)$ in Equation 1 for the same eight groups:

1. Weekday walk trips made by working adults, $Pr\left( p_{m=walk,i} \right)|weekday, working$
2. Weekend walk trips made by working adults, $Pr\left( p_{m=walk,i} \right)|weekend, working$
3. Weekday walk trips made by non-working adults, $Pr\left( p_{m=walk,i} \right)|weekday, nonworking$
4. Weekend walk trips made by non-working adults, $Pr\left( p_{m=walk,i} \right)|weekend, nonworking$
5. Weekday bike trips made by working adults, $Pr\left( p_{m=bike,i} \right)|weekday, working$
6. Weekend bike trips made by working adults, $Pr\left( p_{m=bike,i} \right)|weekend, working$
7. Weekday bike trips made by non-working adults, $Pr\left( p_{m=bike,i} \right)|weekday, nonworking$
8. Weekend bike trips made by non-working adults, $Pr\left( p_{m=bike,i} \right)|weekend, nonworking$

As before, each of these sets of estimates contains unique values for each possible combination of age (ranging from 18-95), sex (male or female), race (non-Hispanic White, non-Hispanic Black, Hispanic, non-Hispanic Asian, or non-Hispanic other), and, for working adults, mode to work (drive, transit, walk, or bike). Additionally, unique estimates for each trip purpose are included for each set. These estimates are stored in a matrix $\mathbf{TPA}_{\mathbf{prob}}$ containing 19,500 cells (five possible purposes, 78 possible ages, two possible sexes, five possible races, and five possible modes to work, including non-working as a fifth mode).

For our example block, estimates of walk trip purpose probabilities for a non-Hispanic Black adult who takes transit to work across age are provided below (Figure S8):


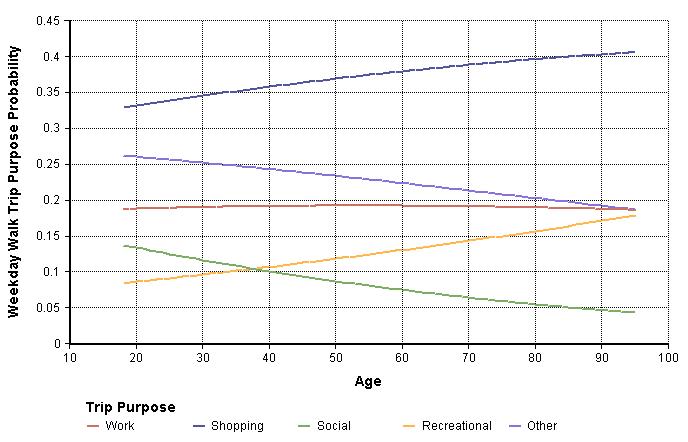


**Figure S8.** Predictions of weekday walk trip purpose probabilities for a non-Hispanic Black working adult who takes transit in work living in the example block group

## Step 3: Estimating walk and bike trip durations for workers and non-workers

Finally, regression coefficients from the GEE models are then used to estimate the duration of walk and bike trips made by an individual for a specific purpose Regression coefficients for these models appear in Table 5 (walk trips) and Table 6 (bike trips). Using the same dimensions as above, these models are used to estimate $\boldsymbol{d}_{\boldsymbol{p}\boldsymbol{,m,i}}$ in Equation 1 for the same eight groups:

1. Weekday walk trips durations for working adults, $\boldsymbol{d}_{\boldsymbol{p}\boldsymbol{,m,i}}|weekday, working$
2. Weekend walk trips durations for working adults, $\boldsymbol{d}_{\boldsymbol{p}\boldsymbol{,m,i}}|weekend, working$
3. Weekday walk trips durations for non-working adults, $\boldsymbol{d}_{\boldsymbol{p}\boldsymbol{,m,i}}|weekday, nonworking$
4. Weekend walk trips durations for non-working adults, $\boldsymbol{d}_{\boldsymbol{p}\boldsymbol{,m,i}}|weekend, nonworking$
5. Weekday bike trips durations for working adults, $\boldsymbol{d}_{\boldsymbol{p}\boldsymbol{,m,i}}|weekday, working$
6. Weekend bike trips durations for working adults, $\boldsymbol{d}_{\boldsymbol{p}\boldsymbol{,m,i}}|weekend, working$
7. Weekday bike trips durations for non-working adults, $\boldsymbol{d}_{\boldsymbol{p}\boldsymbol{,m,i}}|weekday, nonworking$
8. Weekend bike trips durations for non-working adults, $\boldsymbol{d}_{\boldsymbol{p}\boldsymbol{,m,i}}|weekend, nonworking$

As above, each of these sets of estimates contains unique values for each possible combination of trip purpose, age (ranging from 18-95), sex (male or female), race (non-Hispanic White, non-Hispanic Black, Hispanic, non-Hispanic Asian, or non-Hispanic other), and, for working adults, mode to work (drive, transit, walk, or bike). These estimates are stored in a matrix $\mathbf{TPA}_{\mathbf{dur}}$ containing 19,500 cells (five possible purposes, 78 possible ages, two possible sexes, five possible races, and five possible modes to work, including non-working as a fifth mode).

For our example block, estimates of walk trip duration for a non-Hispanic Black adult who takes transit to work across age are provided below (Figure S9):


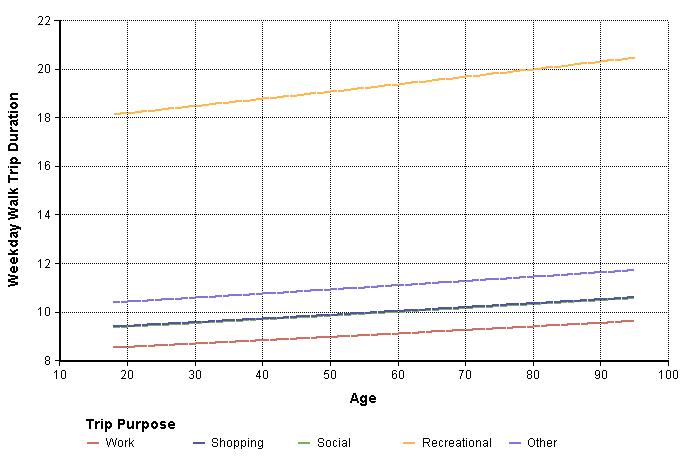


**Figure S9.** Predictions of weekday walk trip durations by purpose for a non-Hispanic Black working adult who takes transit in work living in the example block group

## Step 4: Combing model estimates

Estimates stored in $\mathbf{TPA}_{\mathbf{count}}$, $\mathbf{TPA}_{\mathbf{prob}}$, and $\mathbf{TPA}_{\mathbf{dur}}$, are then combined using Equation 1. For the application included in the main text, durations from recreational trips are not included when calculating Equation 1 (i.e., the summation does not included the fourth purpose, recreational, when summing the product of trip probability and trip duration). This yields the matrix $\mathbf{TPA}_{\mathbf{i}}$ mentioned in-text. The dimensions of this matrix expand as transportation physical activity is estimated for additional block groups.

For our example block, estimates of weekly walk time for a non-Hispanic Black adult across age are provided below (Figure S10):


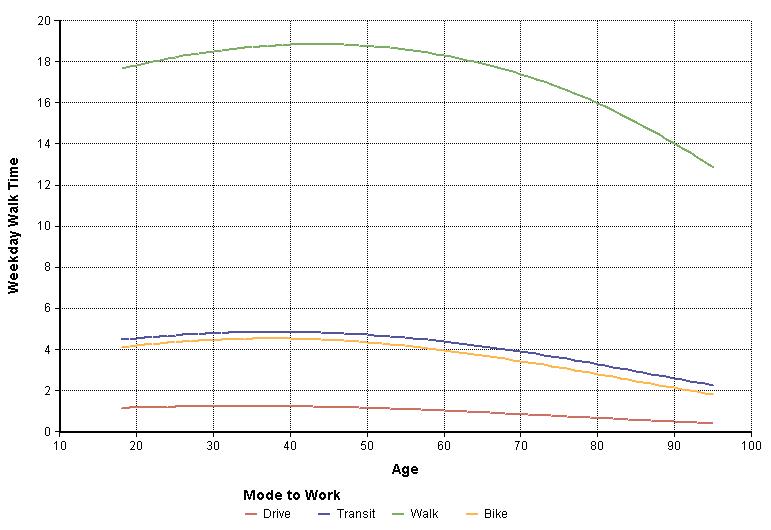


**Figure S10.** Predictions of weekday walking time by commute mode to for a non-Hispanic Black working adult who takes transit in work living in the example block group

## Step 5: Developing a representative population distribution

Transportation physical activity estimates contained in $\mathbf{TPA}_{\mathbf{i}}$ must be applied to a population that is distributed across the same dimensions as the matrix (age, sex, race, mode to work, and block group-level variables). In each block group, cross-tabulations of age and sex are taken from the American Community Survey and used to develop a joint distribution of age and sex in each block group. These data are then multiplied by the distribution of race and commute mode to work, including a category for non-workers, in the block group. Finally, **NPD** is multiplied by the total block group population. This generates a representative population in each block group that has the same dimensions as our transportation physical activity estimates.

In our example block group, the population, distributed by age and sex (Figure S11), is multiplied by the block group distribution of race (Figure S12) and commute mode to work (Figure S13):


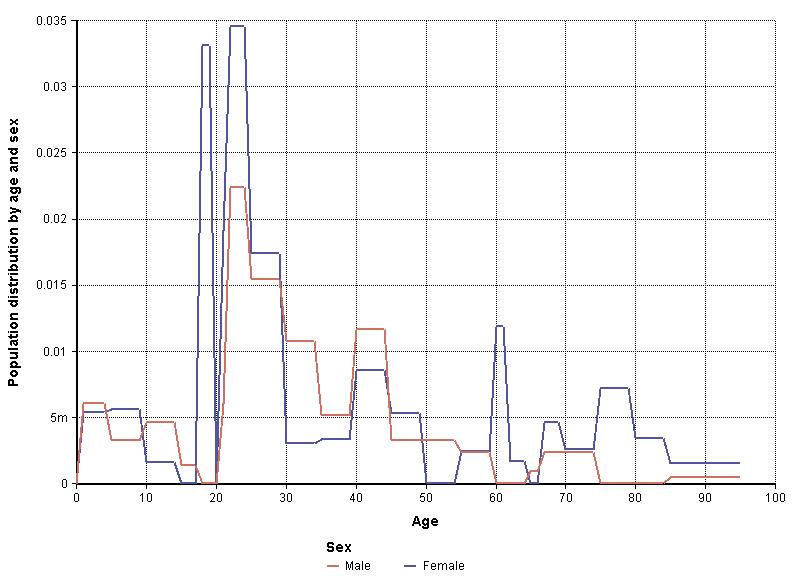


**Figure S11.** Distribution of population for males and females in the example block group


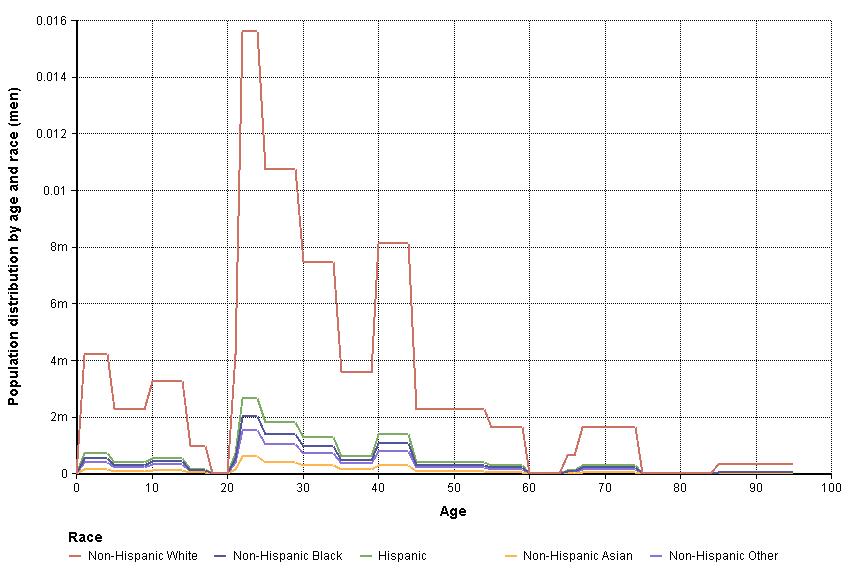


**Figure S12.** Distribution of population race for males in the example block group


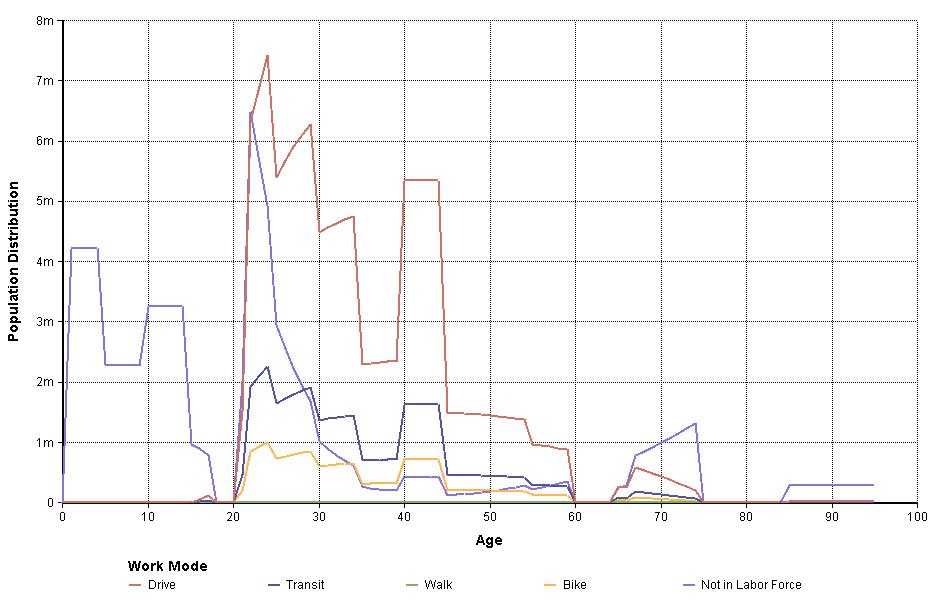


**Figure S13.** Distribution of commute mode to work, including non-workers, for a White male in the example block group

Finally, the distribution above is multiplied by the total block group population to obtain the approximate number of persons in each category of age, sex, race, and mode to work.

## Step 6: Assigning transportation physical activity estimates to the population and estimating health impacts

The representative population in each cell of the matrix storing the population is assigned the corresponding level estimate transportation physical activity stored in $\mathbf{TPA}_{\mathbf{i}}$. In our example, a 50 year old non-Hispanic Black adult who walks is estimated to walk about 105 minutes per week for transportation. This value, plus estimated transportation biking, is transformed to MET-hours using Equation 2. In turn, Equations 6 and 7 are then used, where $f_{est}\left( TPA \right)$ is the distribution of physical activity estimates assigned to the population distribution and $f_{cf}\left( TPA \right)$ is the appropriate counterfactual scenario (Table S7).

**Table S7.** Transportation physical activity levels and estimated health impacts relative to the walkable neighborhood counterfactual for Block Group 2, Census Tract 107.03 in Orange County, North Carolina.

| Commute Mode to Work | Population | Estimate transportation physical activity (MET-hrs/week) | Preventable mortality (deaths/100,000 persons) *^a^* |
| --- | --- | --- | --- |
| Population | 2,142 | 3.39 | -0.89 |
| Drive to work | 856 | 0.85 | 1.69 |
| Transit to work | 261 | 2.97 | -1.02 |
| Walk to work | 0 | n/a | n/a |
| Bike to work | 116 | 26.9 | -30.5 |
| Not in labor force | 909 | 2.47 | 0.50 |
| *^a^* Negative preventable mortality indicates that observed transportation physical activity exceeds the counterfactual scenario and represent existing health benefits relative to the counterfactual (33.7 minutes walking/week) | | | |
