# Supplementary material for: Estimating Active Transportation Behaviors to Support Health Impact Assessment in the United States
Source: Front Public Health. 2016 May 2;4:63. doi: 10.3389/fpubh.2016.00063 (PMC4852202; doi:10.3389/fpubh.2016.00063)
Supplement: Supplementary file 2 [file image_1.PDF]

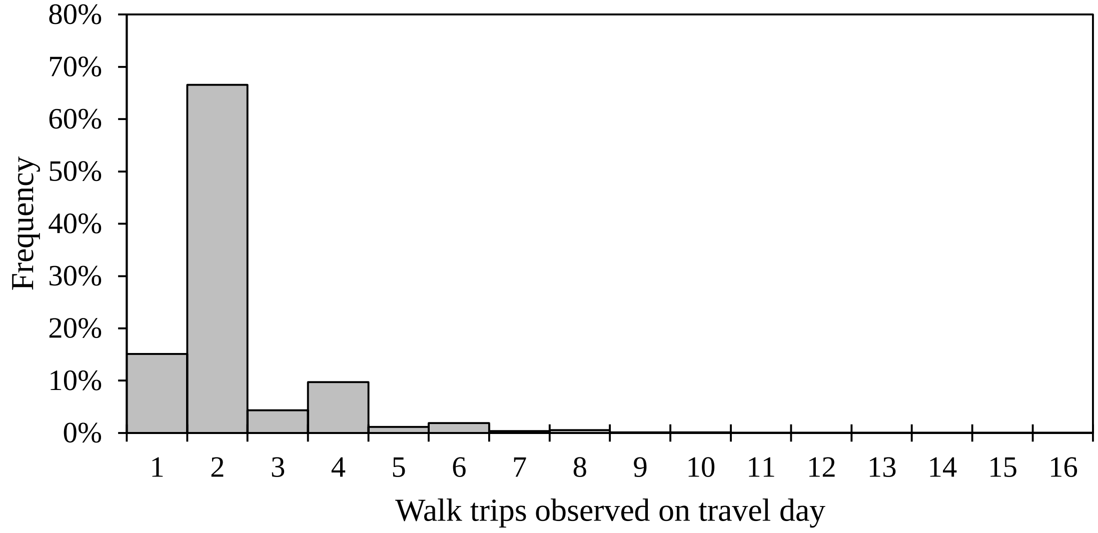

Percent zero counts, travel day walk trips: 86.0%
Mean: 0.32 (including zeroes); 2.27 (excluding zeroes)
Variance: 0.82 (including zeroes); 1.43 (excluding zeroes)

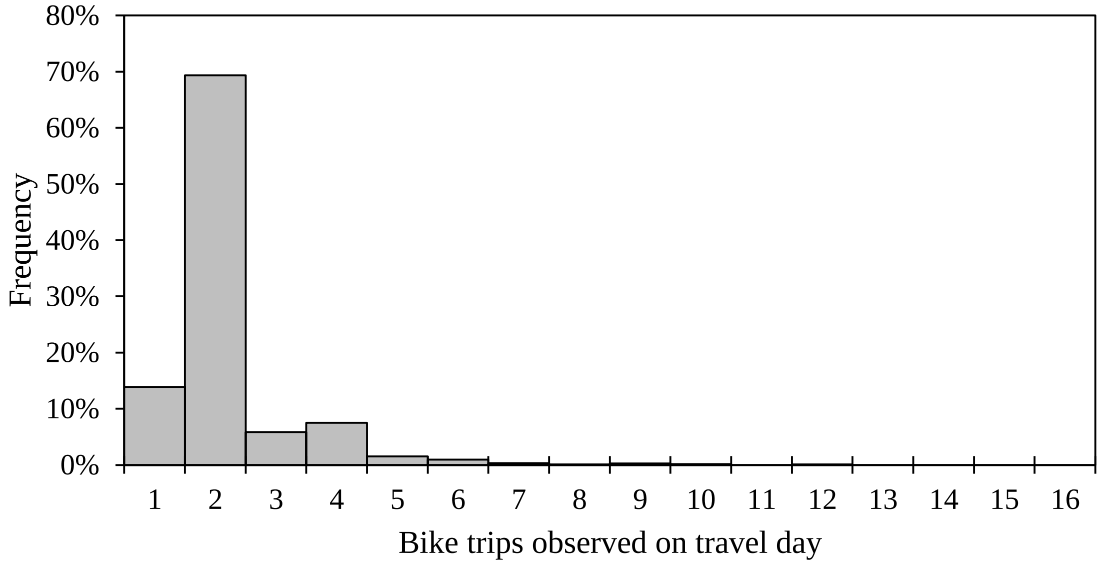

Percent zero, travel day bike trips: 98.9%
Mean: 0.025 (including zeroes); 2.22 (excluding zeroes)
Variance: 0.069 (including zeroes); 1.17 (excluding zeroes)

Figure S1. Distribution of non-zero trip observed walk and bike trips counts and descriptive statistics showing little evidence of overdispersion for non-zero counts
