# Supplementary material for: Estimating Active Transportation Behaviors to Support Health Impact Assessment in the United States
Source: Front Public Health. 2016 May 2;4:63. doi: 10.3389/fpubh.2016.00063 (PMC4852202; doi:10.3389/fpubh.2016.00063)
Supplement: Supplementary file 3 [file image_2.PDF]

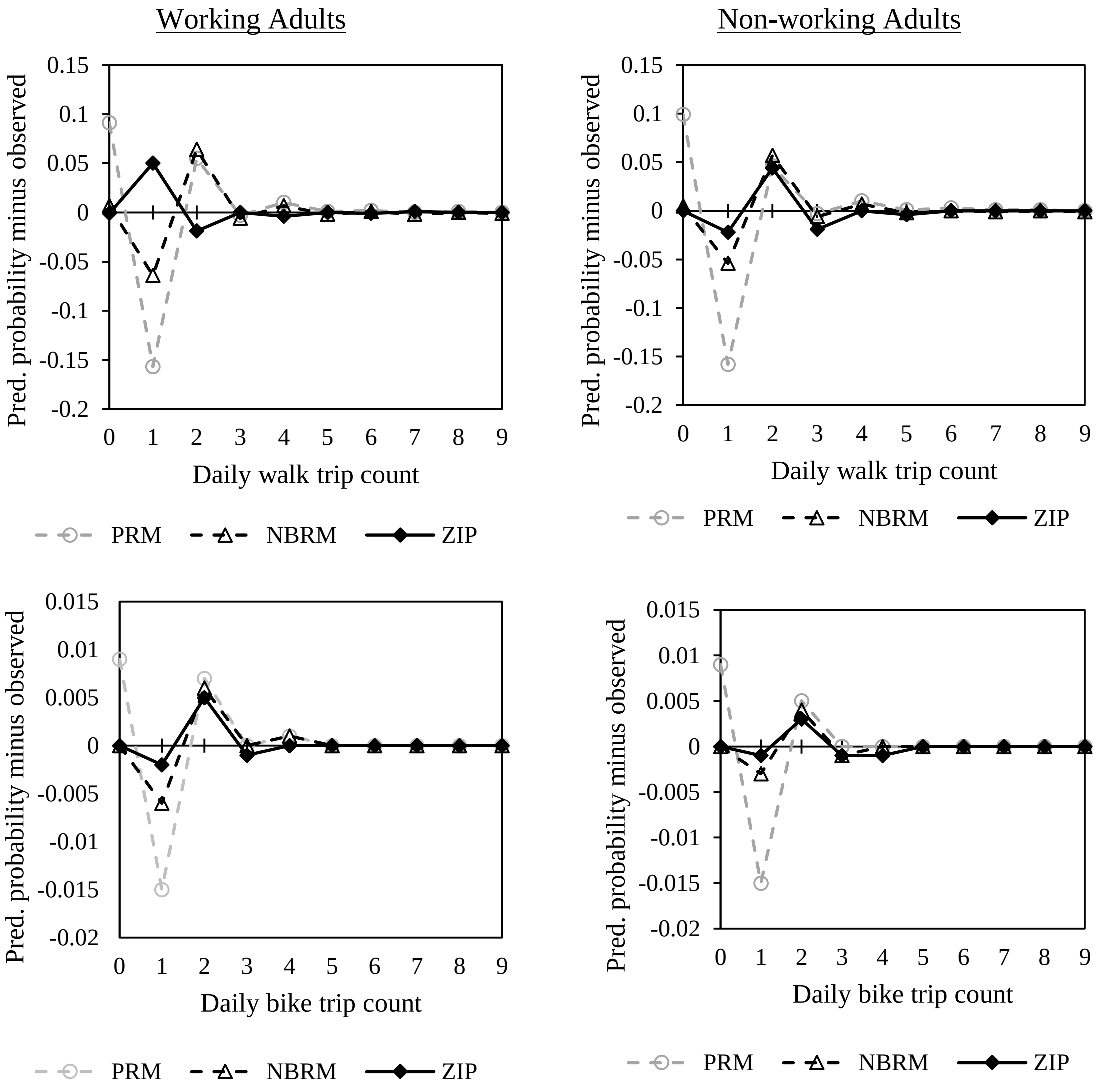

Figure S2. Comparison of model error (predicted probability minus observed) for each model form (Poisson, negative binomial, and zero-inflated Poisson) for walk and bike trip count models for working adults and non-working adults.
