# Supplementary material for: Estimating Active Transportation Behaviors to Support Health Impact Assessment in the United States
Source: Front Public Health. 2016 May 2;4:63. doi: 10.3389/fpubh.2016.00063 (PMC4852202; doi:10.3389/fpubh.2016.00063)
Supplement: Supplementary file 4 [file image_3.PDF]

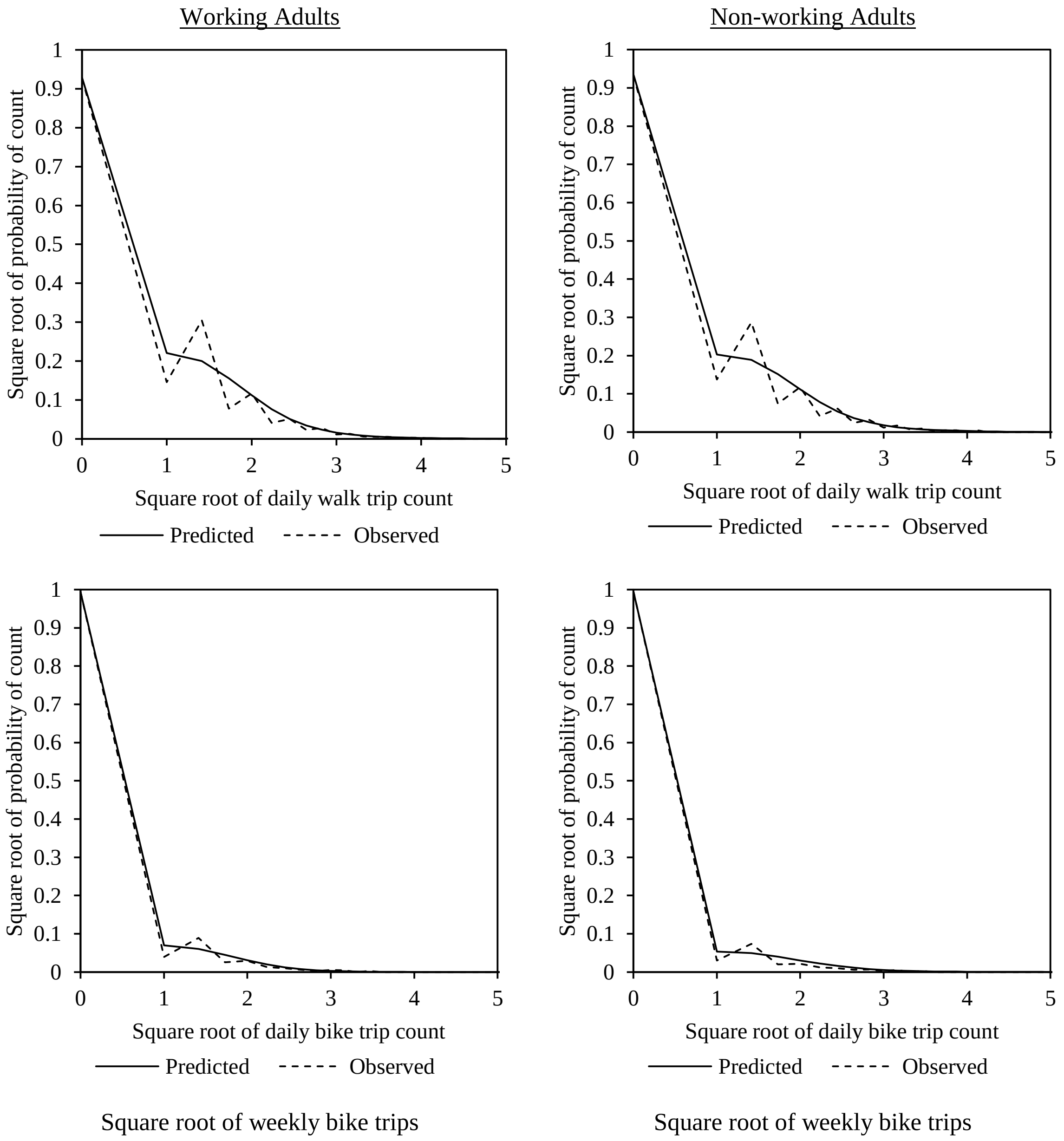

Figure S3. Predicted probabilities of weekly walk and bike trips. Solid black lines illustrate predicted probabilities and observed trip counts are represented by the dashed black line.
