# Supplementary material for: Estimating Active Transportation Behaviors to Support Health Impact Assessment in the United States
Source: Front Public Health. 2016 May 2;4:63. doi: 10.3389/fpubh.2016.00063 (PMC4852202; doi:10.3389/fpubh.2016.00063)
Supplement: Supplementary file 5 [file image_4.PDF]

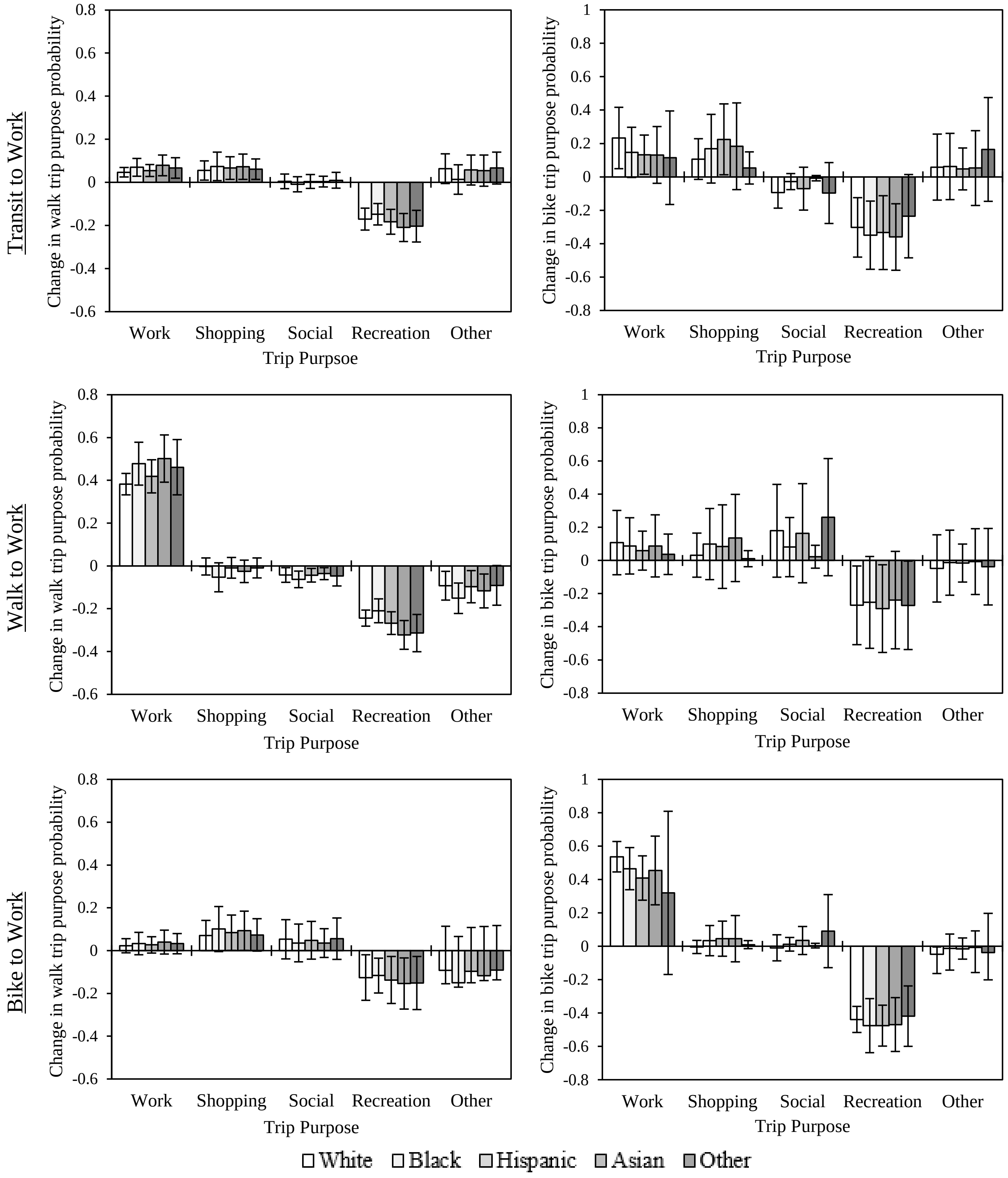

Figure S4. Average marginal effects of commute mode to work on the probability that a given trip is for one of five purposes (listed across the bottom axis) by race/ethnicity relative to the reference group (private automobile to work)
