# Supplementary material for: Estimating Active Transportation Behaviors to Support Health Impact Assessment in the United States
Source: Front Public Health. 2016 May 2;4:63. doi: 10.3389/fpubh.2016.00063 (PMC4852202; doi:10.3389/fpubh.2016.00063)
Supplement: Supplementary file 7 [file image_6.PDF]

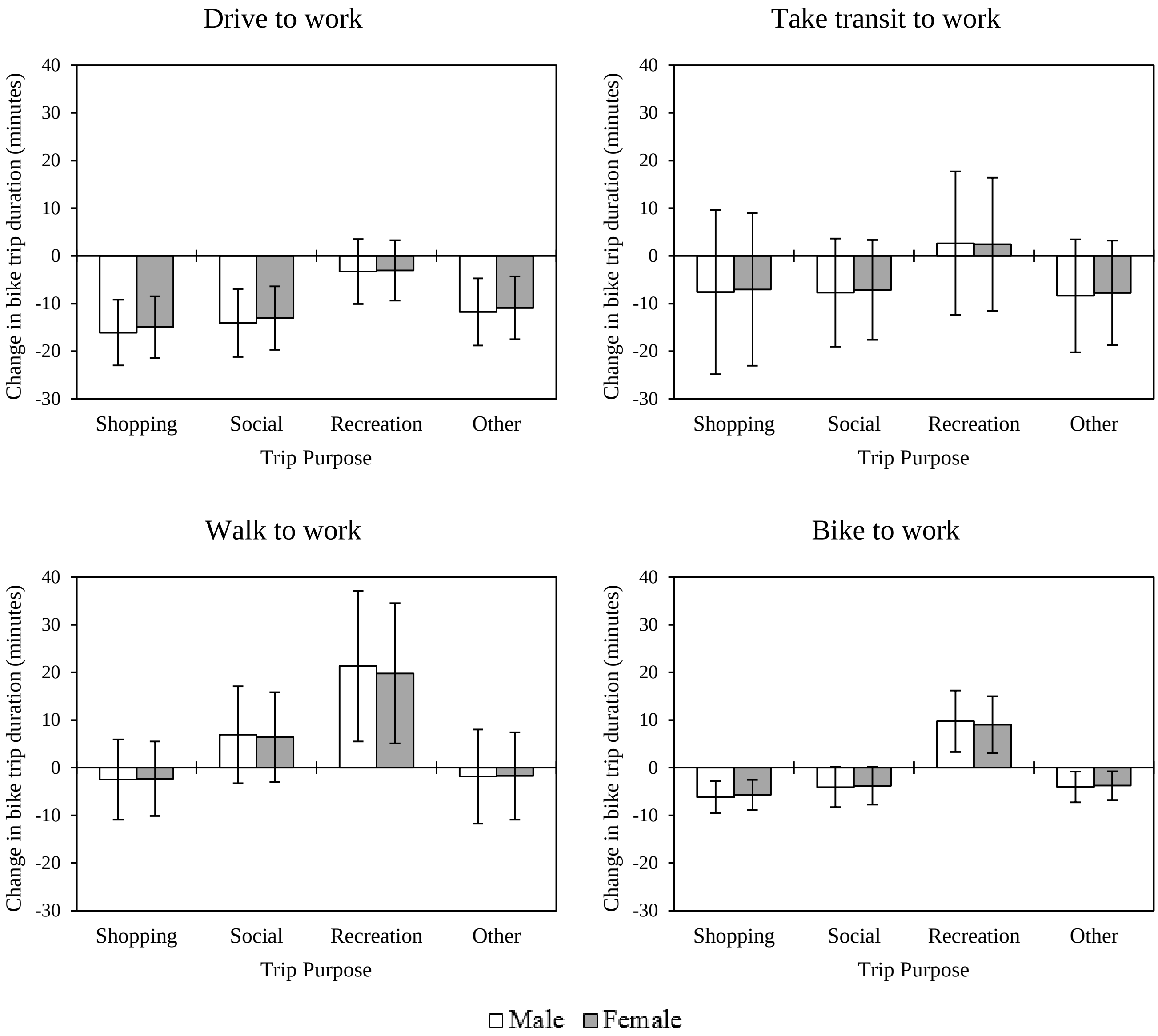

Figure S6. Average marginal effects of trip purpose on bike trip duration for four trip purposes (listed across the bottom axis) relative to work trip duration, by commute mode to work and sex
