# Supplementary material for: Estimating Active Transportation Behaviors to Support Health Impact Assessment in the United States
Source: Front Public Health. 2016 May 2;4:63. doi: 10.3389/fpubh.2016.00063 (PMC4852202; doi:10.3389/fpubh.2016.00063)
Supplement: Supplementary file 8 [file table_1.docx]

**Table S1.** Unweighted Descriptive Statistics, Person Data

|  | |  | 2009 NHTS | | | | 2006 Triangle Survey | |
| --- | --- | --- | --- | --- | --- | --- | --- | --- |
|  | |  | In Labor Force  (n = 109,250) | | Not In Labor Force  (n = 119,743) | | In Labor Force  (n = 3,246) | |
| Variable | | | Mean | S.D. | Mean | S.D. | Mean | S.D. |
| Number of walk trips | | | 0.31 | 0.90 | 0.30 | 0.92 | 0.88 | 3.40 |
| Number of bike trips | | | 0.03 | 0.26 | 0.02 | 0.22 | 0.14 | 0.91 |
| Percentage reporting zero walk trips | | | 86.3% |  | 87.4% |  | 78.8% |  |
| Percentage reporting zero bike trips | | | 98.9% |  | 99.3% |  | 95.7% |  |
| Number of trips on travel day | | | 4.34 | 2.67 | 3.45 | 2.91 | 4.92 | 2.66 |
| Age | | | 87.0 | 13.1 | 64.2 | 16.7 | 47.4 | 13.2 |
| Population density *^a^* | | | 3.55 | 4.99 | 3.46 | 4.96 | 1.56 | 1.63 |
| Percent units rented *^a^* | | | 23.7% | 21.2 | 25.1% | 21.5 | 34.0% | 20.3 |
| Travel time to work *^a^* | | | 23.2 | 17.3 | - | - | 25.9 | 5.36 |
| Mode to work | | |  |  |  |  |  |  |
|  | | Automobile | 95.1% |  | - | - | 94.0% |  |
|  | | Public Transit | 2.53% |  | - | - | 2.50% |  |
|  | | Walk | 1.81% |  | - | - | 2.56% |  |
|  | | Bike | 0.55% |  | - | - | 0.96% |  |
| Male | | | 50.2% |  | 38.9% |  | 42.4% |  |
| Female | | | 49.8% |  | 61.1% |  | 57.6% |  |
| Race/Ethnicity | | |  |  |  |  |  |  |
|  | | Non-Hispanic White | 82.5% |  | 83.8% |  | 82.1% |  |
|  | | Non-Hispanic Black | 5.29% |  | 5.92% |  | 10.9% |  |
|  | | Hispanic | 7.63% |  | 6.73% |  | 3.57% |  |
|  | | Non-Hispanic Asian | 2.72% |  | 1.60% |  | 1.85% |  |
|  | | Non-Hispanic Other | 1.89% |  | 1.90% |  | 1.60% |  |
| Education | | |  |  |  |  |  |  |
|  | | Less than High School | 3.91% |  | 12.0% |  | 2.05% |  |
|  | | High School or GED | 23.8% |  | 32.8% |  | 11.1% |  |
|  | | Some college | 29.5% |  | 27.3% |  | 13.2% |  |
|  | | Bachelor’s/Associate | 24.4% |  | 16.7% |  | 43.3% |  |
|  | | Graduate/Professional | 18.5% |  | 11.3% |  | 30.5% |  |
| Medical Condition | | | 2.69% |  | 23.1% |  | 2.53% |  |
| Heavy Rail in MSA | | | 18.3% |  | 16.3% |  | 0% |  |
| Proxy Respondent | | | 18.2% |  | 16.1% |  | 4.78% |  |
| Season | | |  |  |  |  |  |  |
|  | | Winter | 23.3% |  | 22.6% |  | 68.5% |  |
|  | | Spring | 23.1% |  | 24.8% |  | 0% |  |
|  | | Summer | 27.9% |  | 27.2% |  | 0% |  |
|  | | Fall | 25.6% |  | 25.5% |  | 31.5% |  |
|  | *^a^* For the 2006 Triangle household survey, value is taken from mean value of block group containing household | | | | | | | |
