# Supplementary material for: Estimating Active Transportation Behaviors to Support Health Impact Assessment in the United States
Source: Front Public Health. 2016 May 2;4:63. doi: 10.3389/fpubh.2016.00063 (PMC4852202; doi:10.3389/fpubh.2016.00063)
Supplement: Supplementary file 9 [file table_2.docx]

**Table S2.** Unweighted Descriptive Statistics, Active Trips (NHTS only)

| **Working Adults** | | All active trips  (n = 36,569) | | Walk trips  (n = 33,863) | | Bike trips  (n = 2,706) | |
| --- | --- | --- | --- | --- | --- | --- | --- |
| Variable | | Mean | S.D. | Mean | S.D. | Mean | S.D. |
| Duration (min) | | 14.1 | 12.0 | 13.4 | 10.7 | 22.9 | 20.7 |
| Trip Purpose | |  |  |  |  |  |  |
|  | Work commute | 8.93% |  | 7.54% |  | 26.3% |  |
|  | Shopping | 9.44% |  | 9.38% |  | 10.2% |  |
|  | Social | 9.86% |  | 9.91% |  | 9.13% |  |
|  | Recreational | 36.6% |  | 36.2% |  | 40.9% |  |
|  | Personal/family business | 35.2% |  | 36.9% |  | 13.5% |  |
| Trip duration, by purpose | |  |  |  |  |  |  |
|  | Work commute | 13.3 | 12.9 | 11.0 | 10.2 | 21.2 | 17.5 |
|  | Shopping | 9.86 | 9.56 | 9.44 | 9.08 | 14.7 | 13.0 |
|  | Social | 10.6 | 11.2 | 10.2 | 10.3 | 17.1 | 18.6 |
|  | Recreational | 19.5 | 13.2 | 18.6 | 11.3 | 29.3 | 23.9 |
|  | Personal/family business | 10.9 | 8.7 | 10.7 | 8.33 | 16.8 | 15.6 |
| **Non-working Adults** | | All active trips  (n = 37,311) | | Walk trips  (n = 35,330) | | Bike trips  (n = 1,981) | |
| Variable | | Mean | S.D. | Mean | S.D. | Mean | S.D. |
| Duration (min) | | 15.1 | 12.0 | 14/7 | 11.4 | 21.2 | 19.4 |
| Trip Purpose | |  |  |  |  |  |  |
|  | Work commute | 0% |  | 0% |  | 0% |  |
|  | Shopping | 14.8% |  | 14.7% |  | 16.6% |  |
|  | Social | 15.2% |  | 15.2% |  | 16.5% |  |
|  | Recreational | 43.2% |  | 42.7% |  | 50.5% |  |
|  | Personal/family business | 26.9% |  | 27.5% |  | 16.4% |  |
| Trip duration, by purpose | |  |  |  |  |  |  |
|  | Shopping | 11.8 | 11.0 | 11.6 | 10.8 | 14.0 | 12.9 |
|  | Social | 10.3 | 11.1 | 9.9 | 10.4 | 17.5 | 18.2 |
|  | Recreational | 19.5 | 12.5 | 19.0 | 11.6 | 26.1 | 21.2 |
|  | Personal/family business | 12.4 | 9.83 | 12.3 | 9.52 | 17.2 | 15.9 |
