# Supplementary material for: Estimating Active Transportation Behaviors to Support Health Impact Assessment in the United States
Source: Front Public Health. 2016 May 2;4:63. doi: 10.3389/fpubh.2016.00063 (PMC4852202; doi:10.3389/fpubh.2016.00063)
Supplement: Supplementary file 10 [file table_3.docx]

**Table S3. Walk trip count modes specification tests, from Long and Freese countfit command**

| Model 1: Walk trips, working adults | | | | | | |
| --- | --- | --- | --- | --- | --- | --- |
| PRM | | BIC =145,099 | AIC=144,421 | Prefer | Over | Evidence |
|  | Compared to NBRM | BIC=122,208 | dif=22,891 | NBRM | PRM | Very strong |
|  |  | AIC=121,521 | dif=22,901 | NBRM | PRM |  |
|  |  | LRX2=22,903 | prob=0 | NBRM | PRM | *p=0.000* |
|  | Compared to ZIP | BIC=112,221 | dif=32,878 | ZIP | PRM | Very strong |
|  |  | AIC=110,837 | dif=33,585 | ZIP | PRM |  |
|  |  | Vuong= 97.433 | prob=0 | ZIP | PRM | *p=0.000* |
| NBRM | | BIC =122,208 | AIC=121,521 | Prefer | Over | Evidence |
|  | Compared to ZIP | BIC=112,221 | dif=9,987 | ZIP | NBRM | Very strong |
|  |  | AIC=110,837 | dif=10,684 | ZIP | NBRM |  |
| Model 2: Walk trips, non-working adults | | | | | | |
| PRM | | BIC =152,134 | AIC=151,434 | Prefer | Over | Evidence |
|  | Compared to NBRM | BIC=121,613 | dif=30,621 | NBRM | PRM | Very strong |
|  |  | AIC=120,904 | dif=30,531 | NBRM | PRM |  |
|  |  | LRX2=30,533 | prob=0 | NBRM | PRM | *p=0.000* |
|  | Compared to ZIP | BIC=113,667 | dif=38,467 | ZIP | PRM | Very strong |
|  |  | AIC=112,287 | dif=39,147 | ZIP | PRM |  |
|  |  | Vuong= 93.347 | prob=0 | ZIP | PRM | *p=0.000* |
| NBRM | | BIC =121,613 | AIC=120,902 | Prefer | Over | Evidence |
|  | Compared to ZIP | BIC=113,667 | dif=7,946 | ZIP | NBRM | Very strong |
|  |  | AIC=112,287 | dif=8,617 | ZIP | NBRM |  |
