# Supplementary material for: Estimating Active Transportation Behaviors to Support Health Impact Assessment in the United States
Source: Front Public Health. 2016 May 2;4:63. doi: 10.3389/fpubh.2016.00063 (PMC4852202; doi:10.3389/fpubh.2016.00063)
Supplement: Supplementary file 11 [file table_4.docx]

**Table S4. Bike trip count modes specification tests, from Long and Freese countfit command**

| Model 1: Bike trips, working adults | | | | | | |
| --- | --- | --- | --- | --- | --- | --- |
| PRM | | BIC =19,560 | AIC=19,284 | Prefer | Over | Evidence |
|  | Compared to NBRM | BIC=15,571 | dif=3,989 | NBRM | PRM | Very strong |
|  |  | AIC=15,285 | dif=3,999 | NBRM | PRM |  |
|  |  | LRX2=4,001 | prob=0 | NBRM | PRM | *p=0.000* |
|  | Compared to ZIP | BIC=13,283 | dif=6,278 | ZIP | PRM | Very strong |
|  |  | AIC=12,710 | dif=6,574 | ZIP | PRM |  |
|  |  | Vuong= 25.693 | prob=0 | ZIP | PRM | *p=0.000* |
| NBRM | | BIC =15,571 | AIC=15,285 | Prefer | Over | Evidence |
|  | Compared to ZIP | BIC=13,283 | dif=2,289 | ZIP | NBRM | Very strong |
|  |  | AIC=12,710 | dif=2,575 | ZIP | NBRM |  |
| Model 2: Bike trips, non-working adults | | | | | | |
| PRM | | BIC =19,046 | AIC=18,816 | Prefer | Over | Evidence |
|  | Compared to NBRM | BIC=11,977 | dif=7,069 | NBRM | PRM | Very strong |
|  |  | AIC=11,737 | dif=7,079 | NBRM | PRM |  |
|  |  | LRX2=7,081 | prob=0 | NBRM | PRM | *p=0.000* |
|  | Compared to ZIP | BIC=11,353 | dif=7,692 | ZIP | PRM | Very strong |
|  |  | AIC=10,884 | dif=7,932 | ZIP | PRM |  |
|  |  | Vuong= 23.2 | prob=0 | ZIP | PRM | *p=0.000* |
| NBRM | | BIC =121,613 | AIC=120,902 | Prefer | Over | Evidence |
|  | Compared to ZIP | BIC=11,353 | dif=623 | ZIP | NBRM | Very strong |
|  |  | AIC=10,884 | dif=853 | ZIP | NBRM |  |
