# Supplementary material for: Estimating Active Transportation Behaviors to Support Health Impact Assessment in the United States
Source: Front Public Health. 2016 May 2;4:63. doi: 10.3389/fpubh.2016.00063 (PMC4852202; doi:10.3389/fpubh.2016.00063)
Supplement: Supplementary file 12 [file table_5.docx]

**Table S5. Average marginal effects, daily walk and bike trip count models**

|  |  |  | Mode to Work *(ref: private vehicle)* | | | Population density | Percent rental units |
| --- | --- | --- | --- | --- | --- | --- | --- |
|  |  |  | Public transit | Walk | Bike |  |  |
| Daily walk trips |  | |  |  |  |  |  |
|  | Both Sexes | Non-Hispanic White | 0.49*** | 1.6*** | 0.30*** | 0.006*** | 0.002*** |
|  |  | Non-Hispanic Black | 0.42*** | 1.4*** | 0.24*** | 0.005*** | 0.002*** |
|  |  | Hispanic | 0.47*** | 1.5*** | 0.28*** | 0.006*** | 0.002*** |
|  |  | Non-Hispanic Asian | 0.42*** | 1.4*** | 0.23*** | 0.005*** | 0.002*** |
|  |  | Non-Hispanic Other | 0.47*** | 1.5*** | 0.28*** | 0.006*** | 0.002*** |
| Daily bike trips |  | |  |  |  |  |  |
|  | Male | Non-Hispanic White | 0.06** | 0.004 | 1.4*** | 0.001 | - |
|  |  | Non-Hispanic Black | 0.02** | 0.001 | 0.95*** | 0.0005 | - |
|  |  | Hispanic | 0.04 | 0.003 | 1.5*** | 0.001 | - |
|  |  | Non-Hispanic Asian | 0.01* | 0.001 | 0.93*** | 0.0004* | - |
|  |  | Non-Hispanic Other | 0.05 | 0.004 | 1.4*** | 0.001 | - |
|  | Female | Non-Hispanic White | 0.02 | 0.001 | 0.92*** | 0.0005 | - |
|  |  | Non-Hispanic Black | 0.04* | 0.003 | 1.5*** | 0.001* | - |
|  |  | Hispanic | 0.01* | 0.001 | 0.85*** | 0.0004* | - |
|  |  | Non-Hispanic Asian | 0.03 | 0.002 | 1.2*** | 0.001 | - |
|  |  | Non-Hispanic Other | 0.01 | 0.001 | 0.72*** | 0.0003 | - |
|  | ***p<0.01 **p<0.05 *p<0.10 | | | | | | |
