# Supplementary material for: Estimating Active Transportation Behaviors to Support Health Impact Assessment in the United States
Source: Front Public Health. 2016 May 2;4:63. doi: 10.3389/fpubh.2016.00063 (PMC4852202; doi:10.3389/fpubh.2016.00063)
Supplement: Supplementary file 13 [file table_6.docx]

**Table S6.** Baseline five-year (2009-2013) average death rates per 100,000 persons, by age, sex, and county

| County | Sex | Age Group | | | | | | | | | | | | |
| --- | --- | --- | --- | --- | --- | --- | --- | --- | --- | --- | --- | --- | --- | --- |
|  |  | <1 | 1-4 | 5-9 | 10-14 | 15-19 | 20-24 | 35-34 | 35-44 | 45-54 | 55-64 | 65-74 | 75-84 | 85+ |
| Chatham | Male | 354.5 | 14.4 | 0.0 | 0.0 | 76.5 | 140.1 | 131.8 | 174.4 | 470.3 | 1,031.8 | 1,733.1 | 4,289.9 | 13,368.2 |
|  | Female | 456.2 | 0.0 | 9.9 | 0.0 | 0.0 | 59.1 | 75.4 | 96.1 | 206.3 | 534.2 | 1,072.3 | 3,649.4 | 12,441.1 |
| Durham | Male | 496.7 | 33.7 | 18.4 | 12.4 | 73.5 | 91.4 | 128.6 | 219.0 | 481.4 | 1,107.5 | 2,379.3 | 5,499.7 | 14,403.9 |
|  | Female | 426.2 | 26.2 | 15.7 | 18.6 | 26.6 | 32.0 | 38.7 | 123.2 | 307.0 | 636.1 | 1,521.4 | 4,099.7 | 12,570.4 |
| Franklin | Male | 423.7 | 42.5 | 18.9 | 27.5 | 90.0 | 184.1 | 168.0 | 276.8 | 545.9 | 1,168.8 | 2,860.8 | 6,321.0 | 13,481.2 |
|  | Female | 423.5 | 44.1 | 9.7 | 9.8 | 20.0 | 81.0 | 62.9 | 143.0 | 427.2 | 740.8 | 1,633.9 | 3,973.8 | 12,403.4 |
| Granville | Male | 533.0 | 18.1 | 10.6 | 10.6 | 100.9 | 152.7 | 164.5 | 225.5 | 556.6 | 1,092.5 | 2,513.4 | 6,048.7 | 14,508.5 |
|  | Female | 269.3 | 88.6 | 11.5 | 11.3 | 21.9 | 43.0 | 33.3 | 155.3 | 334.6 | 794.2 | 1,702.6 | 4,741.0 | 12,582.4 |
| Harnett | Male | 574.7 | 30.0 | 21.3 | 48.2 | 115.3 | 183.9 | 169.5 | 274.3 | 609.1 | 1,385.1 | 2,859.2 | 6,580.1 | 15,711.1 |
|  | Female | 437.1 | 6.2 | 13.4 | 9.1 | 64.3 | 37.9 | 60.7 | 129.0 | 351.6 | 722.5 | 1,762.1 | 4,626.9 | 14,329.7 |
| Johnston | Male | 416.5 | 26.5 | 14.3 | 11.4 | 88.4 | 171.1 | 144.1 | 228.4 | 493.0 | 1,260.5 | 2,855.3 | 6,427.9 | 17,881.1 |
|  | Female | 409.8 | 51.7 | 6.1 | 3.2 | 34.4 | 83.5 | 68.2 | 120.8 | 370.4 | 696.9 | 1,741.7 | 4,523.0 | 15,120.2 |
| Nash | Male | 615.7 | 39.8 | 0.0 | 35.8 | 112.1 | 193.0 | 210.0 | 293.6 | 619.1 | 1,426.1 | 2,756.7 | 7,005.8 | 16,910.1 |
|  | Female | 479.0 | 10.1 | 6.6 | 6.1 | 49.1 | 30.9 | 108.0 | 200.7 | 416.2 | 781.5 | 1,753.1 | 4,374.1 | 14,524.7 |
| Orange | Male | 386.1 | 17.2 | 5.0 | 19.0 | 37.6 | 51.1 | 92.0 | 112.2 | 359.8 | 744.4 | 1,692.7 | 5,150.4 | 16,231.0 |
|  | Female | 209.5 | 17.5 | 10.4 | 14.4 | 27.1 | 14.8 | 71.8 | 90.7 | 228.9 | 482.8 | 1,184.5 | 3,842.7 | 13,697.5 |
| Person | Male | 557.9 | 96.5 | 15.2 | 0.0 | 45.5 | 132.4 | 187.8 | 264.9 | 631.5 | 1,172.2 | 2,853.2 | 6,489.5 | 17,165.2 |
|  | Female | 358.2 | 24.4 | 17.0 | 15.5 | 32.5 | 42.6 | 108.0 | 144.2 | 448.9 | 692.5 | 1,668.9 | 4,604.0 | 14,827.5 |
| Wake | Male | 448.7 | 25.8 | 16.6 | 12.5 | 46.2 | 98.9 | 86.5 | 126.3 | 309.0 | 770.9 | 1,744.4 | 5,247.7 | 15,217.2 |
|  | Female | 406.0 | 24.1 | 9.5 | 9.0 | 18.9 | 29.8 | 39.6 | 79.4 | 195.3 | 490.8 | 1,259.9 | 3,867.5 | 13,311.6 |
