# Supplementary material for: Estimating Active Transportation Behaviors to Support Health Impact Assessment in the United States
Source: Front Public Health. 2016 May 2;4:63. doi: 10.3389/fpubh.2016.00063 (PMC4852202; doi:10.3389/fpubh.2016.00063)
Supplement: Supplementary file 14 [file table_7.docx]

# Table S7. Transportation physical activity levels and estimated health impacts relative to the walkable neighborhood counterfactual for block group Block Group 2, Census Tract 107.03 in Orange County, North Carolina.

| Commute Mode to Work | Population | Estimate transportation physical activity (MET-hrs/week) | Preventable mortality (deaths/100,000 persons) *^a^* |
| --- | --- | --- | --- |
| Population | 2,142 | 3.39 | -0.89 |
| Drive to work | 856 | 0.85 | 1.69 |
| Transit to work | 261 | 2.97 | -1.02 |
| Walk to work | 0 | n/a | n/a |
| Bike to work | 116 | 26.9 | -30.5 |
| Not in labor force | 909 | 2.47 | 0.50 |
| *^a^* Negative preventable mortality indicates that observed transportation physical activity exceeds the counterfactual scenario and represent existing health benefits relative to the counterfactual (37.4 minutes walking/week) | | | |
